# Redox Mechanisms, Structural Changes, and Electrochemistry of the Wadsley–Roth Li_*x*_TiNb_2_O_7_ Electrode Material

**DOI:** 10.1021/acs.chemmater.3c02003

**Published:** 2023-11-14

**Authors:** Muna Saber, Sesha Sai Behara, Anton Van der Ven

**Affiliations:** † Department of Chemical Engineering, University of California, Santa Barbara, Santa Barbara, California 93106, United States; ‡ Materials Department, University of California, Santa Barbara, Santa Barbara, California 93106, United States

## Abstract

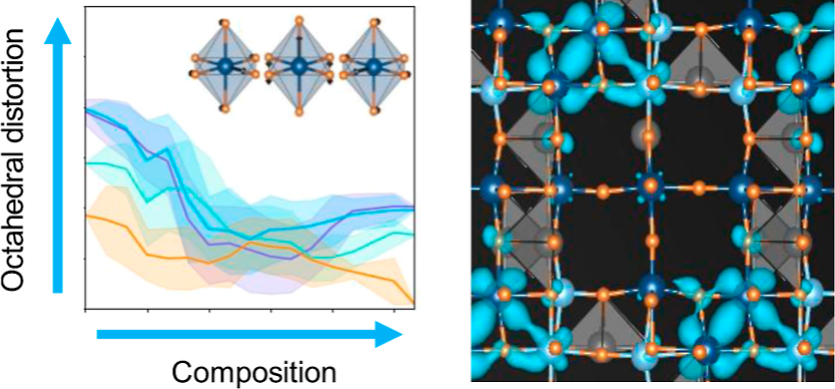

The TiNb_2_O_7_ Wadsley–Roth phase is a promising anode material for Li-ion batteries, enabling fast cycling and high capacities. While already used in commercial batteries, many fundamental electronic and thermodynamic properties of Li_*x*_TiNb_2_O_7_ remain poorly understood. We report on an in-depth first-principles study of the redox mechanisms, structural changes, and electrochemical properties of Li_*x*_TiNb_2_O_7_ as a function of Li concentration. First-principles electronic structure calculations reveal an unconventional redox mechanism upon Li insertion that results in the formation of metal–metal bonds. This metal dimer redox mechanism has important structural consequences as it results in a shortening of cation-pair distances, which in turn affects lattice parameters of the host and thereby alters Li site preferences as the Li concentration is varied. The new insights about redox mechanisms in TiNb_2_O_7_ and their effect on the structure and Li site preferences provide guidance on how the electrochemical properties of a promising class of anode materials can be tailored by exploiting the tremendous structural and chemical diversity of Wadsley–Roth phases.

## 1 Introduction

Lithium-ion batteries continue to dominate the secondary energy storage space. A large fraction of energy storage demand originates in the automotive sector,^1−3^ where electric motors show an efficiency of over 90% and enable the elimination of CO_2_, CO, NO_*x*_, and SO_*x*_ exhaust emitted by internal combustion engines.^4^ Despite their benefits and widespread usage, current lithium-ion battery technologies that rely on graphite anodes possess lower charge rates, power densities, and operating temperature ranges than are needed for future generations of electric vehicles.^5,6^

Wadsley–Roth phases are a family of complex inorganic chemistries that exhibit high power densities when used as electrode materials.^7−19^ The TiNb_2_O_7_ compound is a commercialized Wadsley–Roth phase that can reversibly cycle while achieving a capacity of 340 mA h g^–1^.^16,20^ This compound is also capable of cycling at a rate of 20 C with little gravimetric capacity losses.^21−23^ TiNb_2_O_7_ was first demonstrated to intercalate lithium by Cava in 1983^7^ and was first cycled against lithium in 2011.^11^ TiNb_2_O_7_ has since displayed the capability to supply high power densities at high charge rates.^24,25^

Despite an increased interest in Wadsley–Roth phases as anodes in Li-ion batteries,^14−16,18,26,27^ there is still a limited understanding of the electronic, thermodynamic, and structural properties of these phases as a function of the degree of Li insertion. This paper examines the full lithiation profile of TiNb_2_O_7_ using first-principles statistical mechanics calculations to understand the redox mechanisms and their effect on the structural properties and Li site preferences. We discover an unconventional redox mechanism upon Li insertion into TiNb_2_O_7_ that is not localized on individual transition-metal cations but instead occurs on the bonding states that arise from the hybridization between the t_2g_ orbitals of edge-sharing transition-metal cations. The filling of these bonding states leads to a shortening of metal–metal distances, which has structural consequences for the host more broadly, thereby modifying the Li-site preference as a function of Li concentration. The metal–metal dimer redox mechanism, enabled by crystallographic features shared by all Wadsley–Roth phases, is likely to be relevant for a broad family of promising anode materials for Li-ion batteries.

## 2 Methods

First-principles electronic structure calculations were performed with the density functional theory (DFT) using the generalized gradient approximation (GGA) parametrization of Perdew, Burke, and Ernzerhof (PBE)^28^ as implemented in the Vienna ab initio simulation package (VASP).^29,30^ Several benchmark calculations were also performed with the strongly constrained and appropriately normed (SCAN) meta GGA.^31^ Past studies have shown that SCAN is able to accurately describe the electronic structure of transition-metal oxides in high oxidation states when compared to predictions of the random phase approximation.^32^ Interactions between core and valence electrons were treated with the projector augmented wave (PAW) method.^33,34^ A plane-wave energy cutoff of 650 eV and a reciprocal space discretization of 25 *K*-points per Å^–1^ was used. All calculations were performed spin-polarized with magnetic moments initialized in a ferromagnetic state. The systematic enumeration of different Li-vacancy orderings over the interstitial sites of the TiNb_2_O_7_ Wadsley–Roth crystal structure was performed with the clusters approach to statistical mechanics (CASM)^35,36^ simulation package. Finite temperature electrochemical properties were calculated with CASM by applying Monte Carlo simulations to cluster expansion Hamiltonians trained to a large DFT data set of formation energies.^35,37^

Uncertainty quantification was performed within a Bayesian framework^38−40^ by sampling multiple cluster expansion Hamiltonians from a posterior distribution and calculating electrochemical properties for each sampled cluster expansion.^41^ A standard deviation of 1 meV/atom on the calculated formation energies was used in the Gaussian likelihood distribution, and a standard deviation of 49 meV was assumed for the Gaussian prior distribution of each effective cluster interaction (ECI), the coefficients of the cluster expansion. The mean of the prior distribution of the ECI was chosen to ensure that the predicted ground states and low energy structures are consistent with the DFT predictions as described by Ober et al.^41^

## 3 Results

### 3.1 TiNb_2_O_7_ Wadsley–Roth Host Structure

**Figure 1:**
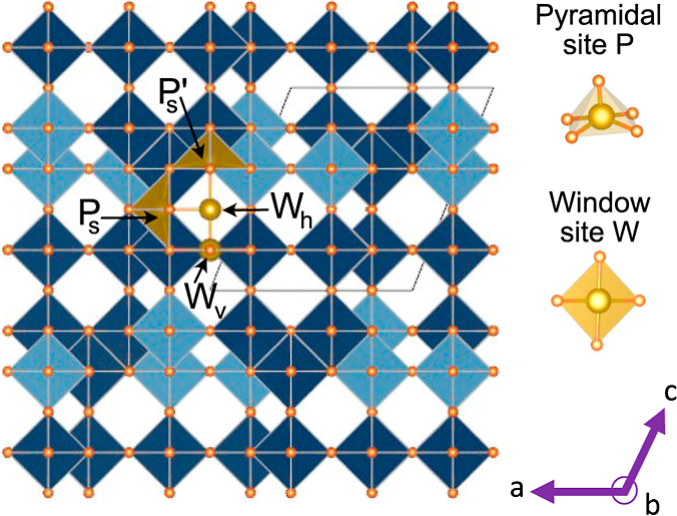
Idealized Wadsley–Roth crystal structure of TiNb_2_O_7_ consisting of corner-sharing and edge-sharing TiO_6_ (light blue) and NbO_6_ (dark blue) octahedra. The Ti and Nb of this model adopt their lowest energy arrangement over the octahedrally coordinated cation sites. The TiO_6_ and NbO_6_ octahedra are highly distorted in the fully relaxed TiNb_2_O_7_ structure (not shown). Intercalated Li ions can occupy pyramidal and window sites.

Wadsley–Roth phases are a family of chemistries with crystal structures derived from the perovskite-like ReO_3_ phase.^7,42^ They consist of infinitely long blocks of *n* × *m* corner-sharing transition-metal oxygen octahedra. The blocks can tile space in different ways with octahedra at the peripheries of each block sharing edges with octahedra of neighboring blocks. Figure 1 shows the Wadsley–Roth structure adopted by TiNb_2_O_7_, which is made of 3 × 3 blocks.

The unit cell of the TiNb_2_O_7_ Wadsley–Roth structure of Figure 1 has nine transition-metal sites. There are a total of 44 symmetrically distinct ways of arranging Ti and Nb over the cation sites of the primitive unit cell of TiNb_2_O_7_. The arrangement with the lowest energy as predicted with DFT-PBE is shown in Figure 1.^15,43^ The higher oxidation state Nb^5+^ cations prefer to occupy the corner-sharing octahedra at the center of the block, while lower oxidation state cations, such as Ti^4+^, tend to segregate to the edge-sharing octahedra at the peripheries of each block. Experimentally synthesized forms of TiNb_2_O_7_ exhibit some degree of disorder among Ti and Nb because the phase is cooled from high temperatures. Nevertheless, neutron diffraction studies have shown that Ti preferentially occupies edge sites, while Nb preferentially occupies the corner-sharing site at the center of the block.^44^ We use the lowest energy Ti–Nb ordering of Figure 1 as a model to explore redox mechanisms and Li-site preferences upon Li insertion.

The TiNb_2_O_7_ Wadsley–Roth crystal can host Li-ions in four types of interstitial sites coordinated by oxygen.^15^ These are shown in Figure 1. Two groups of interstitial sites are pyramidally coordinated by five oxygen ions. These are termed P_s_ and P_s_′. They differ by the number of transition-metal cations that share edges with the sites; the P_s_ sites share six edges with the neighboring transition metals while the P_s_′ sites share seven. There are six P_s_ sites and two P_s_′ sites within each unit cell of TiNb_2_O_7_. The TiNb_2_O_7_ host can also accommodate Li in horizontal and vertical window sites, labeled W_h_ and W_v_, respectively. Both sites are coordinated by four oxygen ions in a square planar configuration. There are four W_h_ and four W_v_ sites per unit cell of TiNb_2_O_7_ residing in the blocks, as illustrated in Figure 1.

### 3.2 Li Insertion into TiNb_2_O_7_ at Zero Kelvin

**Figure 2:**
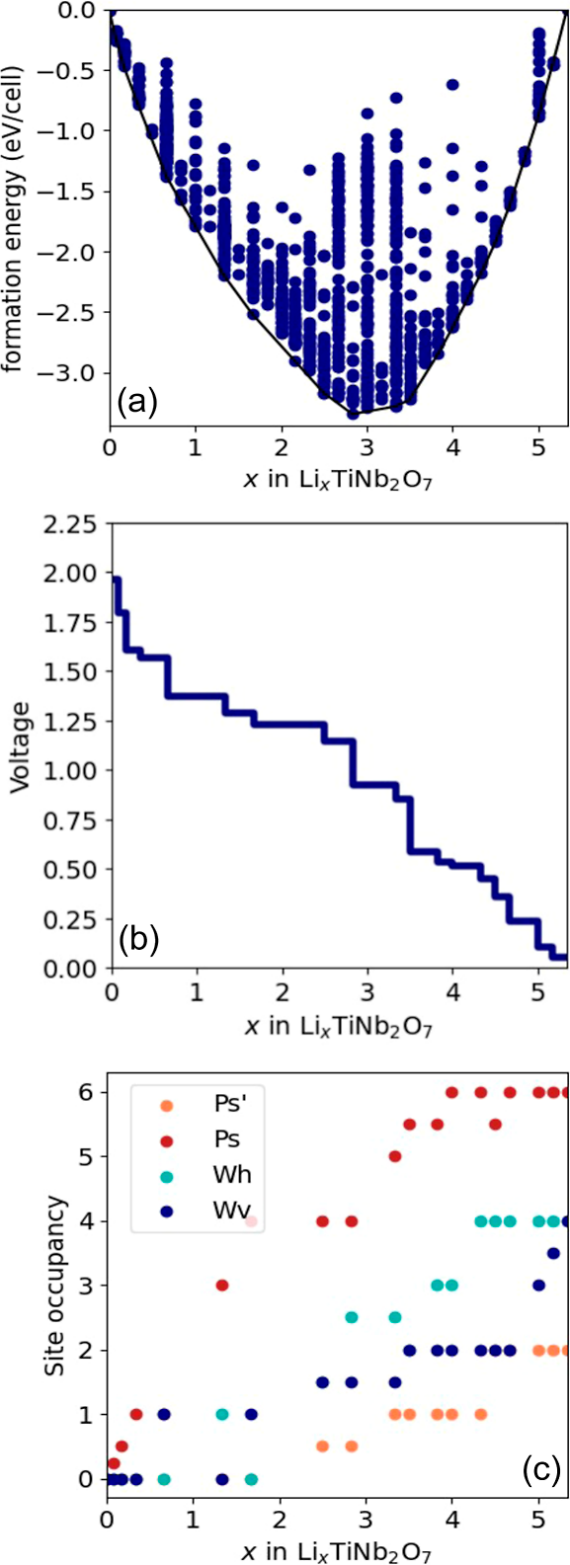
(a) Formation energies of Li_*x*_TiNb_2_O_7_ of 937 Li-vacancy orderings over the pyramidal and window sites of TiNb_2_O_7_ having the Ti–Nb ordering of Figure 1. (b) 0 K voltage curve as a function of composition for Li_*x*_TiNb_2_O_7_. (c) Lithium occupancy of the pyramidal and window sites of Li_*x*_TiNb_2_O_7_ in the ground-state orderings.

The energies of 937 symmetrically distinct orderings of Li and vacancies over the interstitial sites of TiNb_2_O_7_ (i.e., P_s_, P_s_′, W_v_ and W_h_) were calculated with DFT-PBE. In each of these structures, the TiNb_2_O_7_ host has the lowest energy Ti–Nb ordering, as shown in Figure 1. The different Li-vacancy configurations were enumerated with CASM^36^ and include different orderings within the primitive unit cell and in super cells, in which the *b* axis along the block lengths are doubled or quadrupled. Figure 2a shows the calculated formation energies as a function of the Li concentration. Many Li-vacancy arrangements reside on the convex hull. However, with the exception of three ordered phases at *x* = 0.66, *x* = 2.833, and *x* = 3.5, most are weakly stable and very close in energy to other structures that have similar Li-vacancy arrangements and compositions.

The voltage at 0 K is linearly related to the slope of the convex hull as a function of lithium concentration.^37^ The 0 K voltage profile is shown in Figure 2b. Each ordered phase with an energy on the convex hull appears as a step in the 0 K voltage curve, while each plateau corresponds to a two-phase coexistence between a pair of ordered phases.^37,45,46^ The voltage curve, which starts at 2 V upon insertion of a dilute concentration of Li, shows that TiNb_2_O_7_ can intercalate more than 5 Li per formula unit, while maintaining a positive voltage.

An important property affecting Li diffusion is the Li site preference as a function of Li concentration.^15,47−49^Figure 2c plots the number of each type of Li site that is occupied in each ground-state ordering. The pyramidal P_s_ sites fill initially and have the highest occupancy at all Li concentrations. For the Ti and Nb ordering shown in Figure 1, the vertical window sites begin to fill at *x* = 0.66. Both the vertical and horizontal window sites, W_v_ and W_h_, fill steadily beyond *x* = 0.66, but the largest fraction of Li continue to fill the pyramidal P_s_ sites until they are close to saturated at around *x* = 3.5. Close to half the vertical window sites are filled beyond *x* = 2.5, which is consistent with neutron diffraction studies.^50^ The pyramidal P_s_′ sites, which share seven edges with neighboring transition-metal cations, only start to fill gradually past *x* = 2.

### 3.3 Redox Mechanisms: Formation of Metal–Metal Bonds

**Figure 3:**
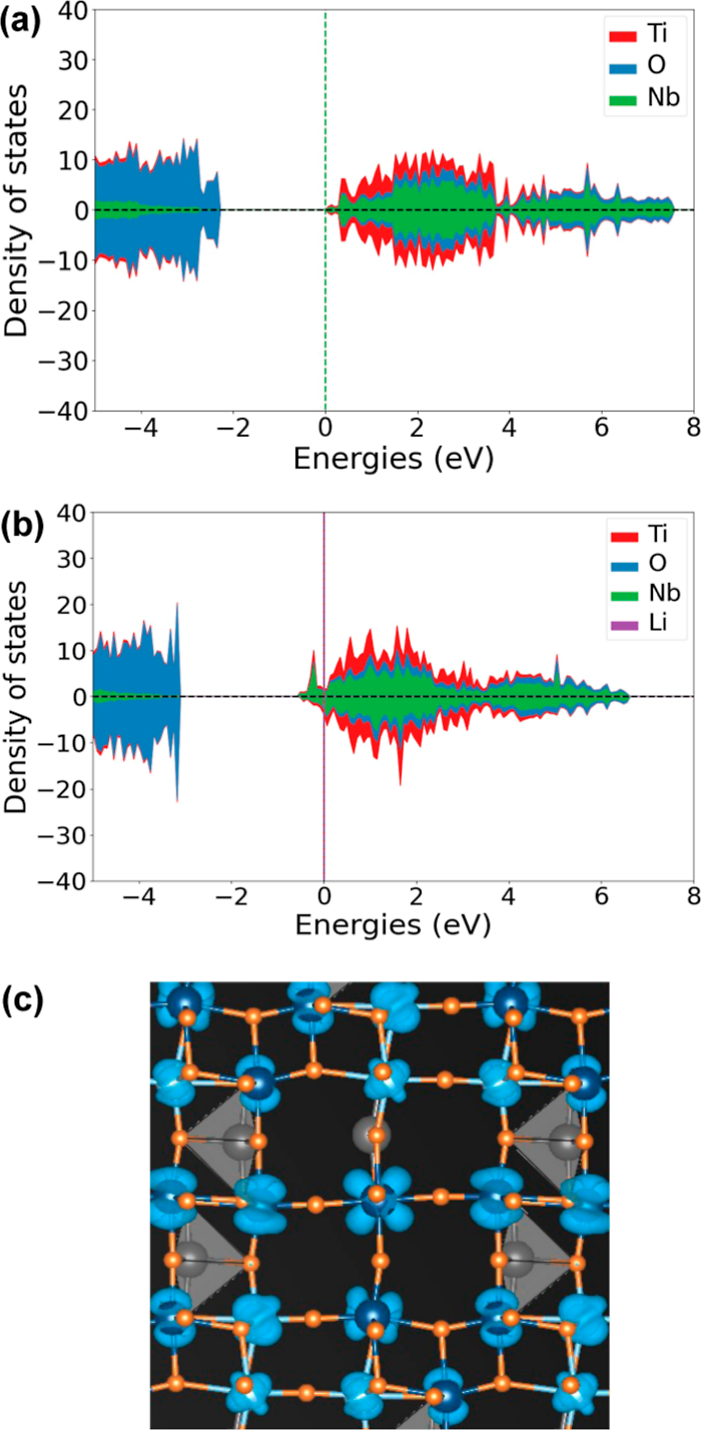
Electronic density of states (DOS) of (a) TiNb_2_O_7_ and (b) LiTiNb_2_O_7_ as calculated with DFT-PBE. The positive (negative) DOS corresponds to spin up (down) states. (c) Electronic charge density corresponding to the filled states in the conduction band of LiTiNb_2_O_7_.

The pristine TiNb_2_O_7_ Wadsley–Roth phase is an insulator. At this composition, the d^0^ Ti^4+^ and Nb^5+^ cations are in their maximum oxidation states. Figure 3a, shows that DFT-PBE predicts a large band gap in the electronic density of states (DOS) of TiNb_2_O_7_. The gap separates the filled valence bands, which primarily have an oxygen *p* character, and the bottom of the empty conduction bands, which are derived from the t_2g_ orbitals of the octahedrally coordinated transition metals (i.e., the d_*xy*_, d_*xz*_, and d_*yz*_ orbitals in an octahedral environment). This is consistent with past calculations by Catti et al.^50^ and Griffith et al.^15^

The addition of Li to TiNb_2_O_7_ leads to a reduction of the formal oxidation states of the Ti and Nb cations. At low Li concentrations, DFT-PBE predicts that the electrons donated to the host by Li tend to delocalize over the different transition metals, with a slight enhancement in the occupancy of the d_*xy*_ orbital on the central Nb site.^15,51^ This is shown in Figure 3b for LiTiNb_2_O_7_. There is some degree of spin polarization, with the spin up DOS (positive) having more states below the Fermi level than the spin down DOS (negative). Figure 3b shows that the donated electrons fill the bottom of the conduction band without substantially altering the DOS of the conduction bands of pristine TiNb_2_O_7_. Figure 3c plots the electronic charge density for the states between the bottom of the conduction band and the Fermi level of LiTiNb_2_O_7_. The electronic charge density concentrates around the transition-metal cations and adopts the characteristic charge density distribution of t_2g_ orbitals (i.e., d_*xy*_, d_*xz*_, and d_*yz*_).

**Figure 4:**
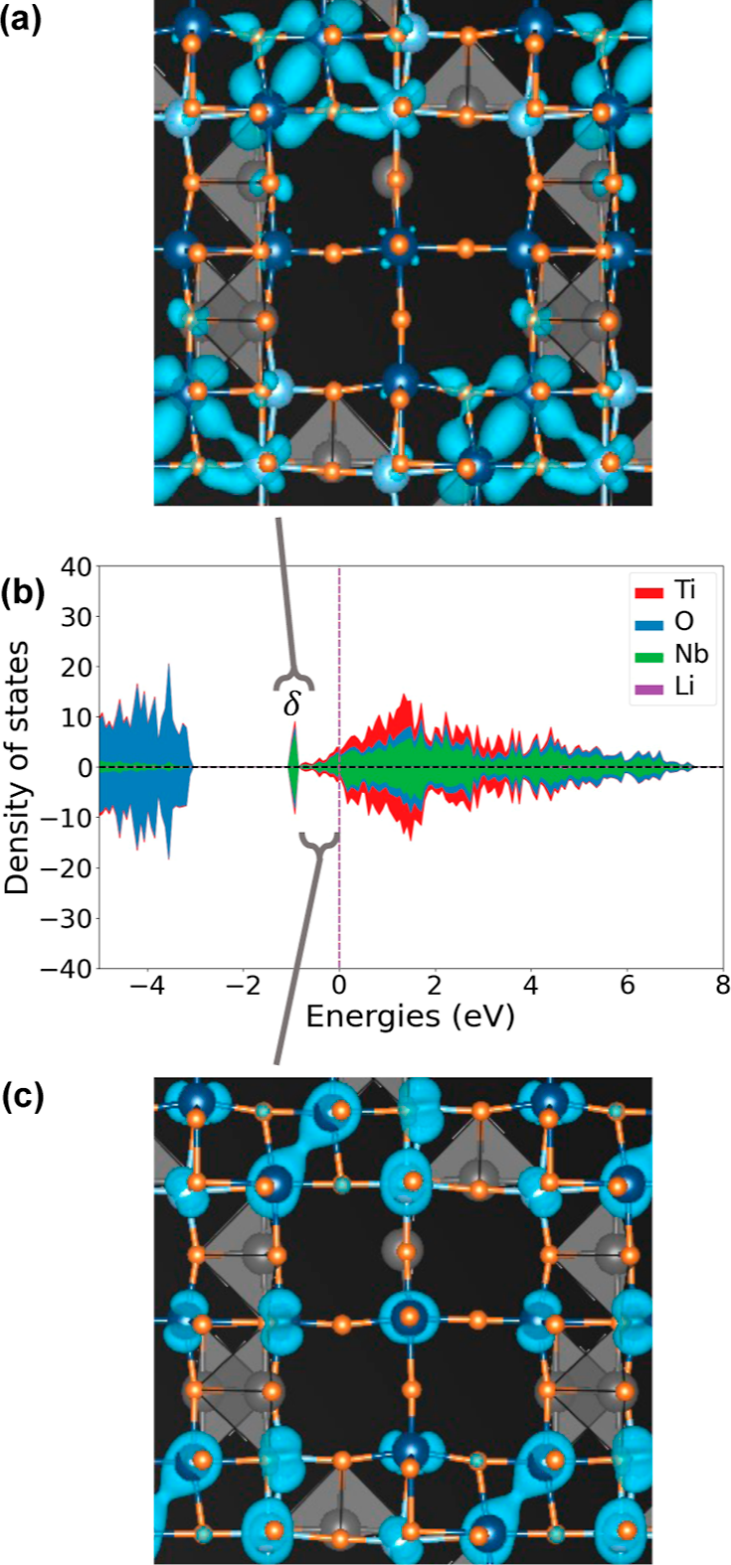
(a) Electronic charge density corresponding to the peaks labeled δ in the DOS of Li_1.66_TiNb_2_O_7_ shown in (b). (c) Electronic density of states for the remaining filled states below the Fermi level.

The redox mechanism changes qualitatively with increasing Li concentration beyond *x* = 1. This is evident in Figure 4 for Li_1.66_TiNb_2_O_7_. New states emerge below the Fermi level (labeled δ in Figure 4b) that are disconnected from the more itinerant bands derived from the t_2g_ states. Figure 4a plots the electronic charge density due to the breakaway peaks in the DOS of Li_1.66_TiNb_2_O_7_ (Figure 4b), clearly showing that the charge density associated with these states is concentrated between a pair of edge-sharing Nb cations. An analysis of the local projected density of states of each of the two Nb atoms of the pair shows that their d_*xy*_ orbitals have energies that coincide with the peaks below the Fermi level in Figure 4b. The electronic charge density due to the remaining DOS that extends from the top of the breakaway peaks up to the Fermi level is shown in Figure 4c. These states have a combined electronic charge density that is more uniformly distributed throughout the crystal and more centered around individual transition-metal cations than between metal cations.

**Figure 5:**
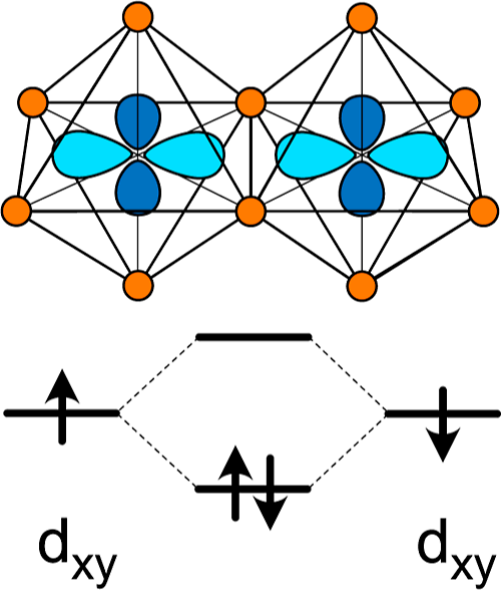
d_*xy*_ orbitals of edge-sharing transition-metal cations can hybridize to form bonding and antibonding states. The bonding states have a lower energy than the unhybridized d_*xy*_ states and can host two electrons of opposite spin.

The enhanced electron charge density between the pair of edge-sharing Nb in Figure 4a is consistent with a filled bonding state that arises when the d_*xy*_ orbitals of a pair of edge-sharing transition-metal cations hybridize, as schematically illustrated in Figure 5.^52−54^ The hybridization between edge-sharing d_*xy*_ orbitals leads to metal–metal dimer formation and generates bonding states that have a lower energy than the unhybridized d_*xy*_ orbitals.^52^ The bonding states are therefore favorable redox centers to accommodate the electrons donated by Li to the host. It is clear in the DOS of Figure 4b that a pair of electrons with opposite spin fills the bonding state. The filling of the bonding state of a hybridized metal–metal bond will lead to a shortening of the distance between the neighboring transition-metal cations.^54^ This is indeed predicted to occur, with the distance between the pair of Nb cations that form a metal-metal bond contracting from a value of 3.09 Å at *x* = 1 to 2.69 Å at *x* = 1.66.

**Figure 6:**
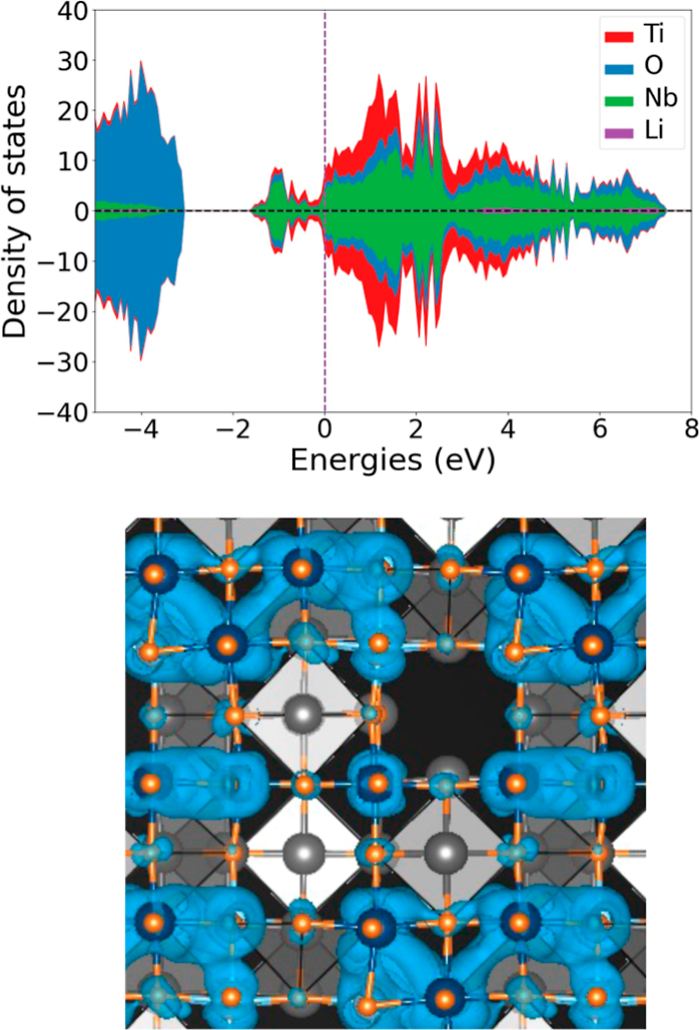
Electronic DOS of Li_2.5_TiNb_2_O_7_ and electronic charge density of states extending from the bottom of the conduction band up to the Fermi level.

Additional metal–metal bonds form between edge-sharing octahedra, as electrons are added to Li_*x*_TiNb_2_O_7_ upon further insertion of Li. At *x* = 2.5, for example, a complex of metal–metal bonds become evident in the charge density plot of Figure 6. Each edge-sharing pair of transition-metal cations with an enhanced charge density along the bond axis also has a shortened metal–metal distance, which is consistent with the filling of the bonding states that arises from metal–metal dimer formation. Several transition-metal cations even participate in two metal–metal bonds. A transition metal with two edge-sharing cation neighbors can form a separate bond with each neighbor with orthogonal onsite t_2g_ orbitals.^54^

TiNb_2_O_7_ starts out as an insulator, but its electronic conductivity increases dramatically upon the addition of Li.^15^ This is consistent with the calculated electronic density of states plots of Figures 3a,b, 4b, and 6b, which show a sizable electronic density of states at the Fermi-level. This suggests the presence of itinerant electrons and more metallic behavior. Even in the presence of localized metal–metal bonds, which have electronic densities of states below the Fermi level (Figures 4b and 6b), there continues to be a large density of states at the Fermi level corresponding to more itinerant electrons.

**Figure 7:**
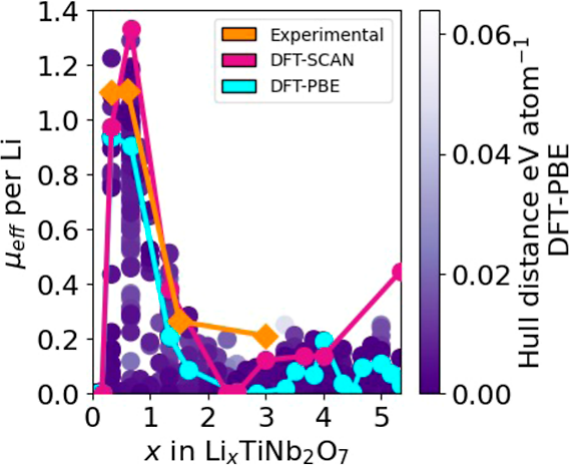
Magnetic moments normalized by the number of Li ions of 937 Li_*x*_TiNb_2_O_7_ structures as calculated with DFT-PBE. The magnetic moments per Li of the ground-state structures are connected by the blue line. The red points are the magnetic moments per Li of the ground-state structures as calculated with DFT-SCAN. Orange points are magnetic moments per Li as measured by Griffith et al.^15^

While the DOS plot of Figure 3 shows that there is some degree of spin polarization at *x* = 1, the DOS plots of Figures 4 and 6 show that the filled bonding states of the metal–metal dimers are nonspin polarized. Figure 7 collects the calculated magnetic moments of all 937 Li_*x*_TiNb_2_O_7_ structures normalized by the number of Li (i.e., number of electrons donated to the host) as a function of Li concentration. The magnetic moments are expressed in units of a Bohr magneton μ_B_ and the numerical values plotted in Figure 7 are calculated as the difference in number of up spin and down spin electrons per Li multiplied by , where *g* = 2 and *s* = 1/2. The light blue line connects the magnetic moment per Li for the ground states of Li_*x*_TiNb_2_O_7_. Figure 7 shows that at dilute concentrations the electrons donated to the host adopt a spin-polarized configuration, resulting in a net magnetic moment of the crystal. However, above *x* = 1, the magnetic moment per Li ion decreases to negligible values. It is above this concentration that the bonding states of the metal–metal dimers are predicted to accommodate the electrons donated by Li to the host. Figure 7 also shows the magnetic moment per Li (in units of μ_B_) as measured experimentally by Griffith et al.^15^ The qualitative agreement between the calculated and measured magnetic moments per Li ion is very good. For comparison, Figure 7 also shows the calculated magnetic moment per Li in the ground-state configurations as calculated with DFT-SCAN. The same trend is predicted, and the values are similar to those predicted with DFT-PBE.

### 3.4 Effect of Redox Mechanism on Structure

The pristine TiNb_2_O_7_ crystal structure has highly distorted MO_6_ octahedra (M = Ti or Nb) due to the oxidation states of Ti and Nb and the large number of edge-sharing MO_6_ octahedra. The d^0^ Ti^4+^ and Nb^5+^ cations are susceptible to second-order Jahn–Teller distortions when octahedrally coordinated by oxygen.^55,56^ This causes displacement of the cations away from the center of their coordinating octahedra. The edge-sharing MO_6_ octahedra of TiNb_2_O_7_ also undergo significant distortions due to the strong electrostatic repulsion between neighboring Ti^4+^ and Nb^5+^ cations.^43^ This repulsion increases the distance between edge-sharing cations, causing a further off-centering of each cation that simultaneously induces collateral distortions of their surrounding oxygen octahedron.^43^

The donation of electrons to the host upon Li insertion undoes many of the octahedral distortions that are initially present in TiNb_2_O_7_. The reduction of the Ti^4+^ and Nb^5+^ cations eliminates their susceptibility to second-order Jahn–Teller distortions. Furthermore, the redox mechanism described in the previous section, which leads to metal–metal dimer formation, has structural consequences that affect the lattice parameters of the host. Each edge-sharing pair of transition metal cations that hybridize to form metal–metal dimers undergo a contraction that pulls the cations back toward the centers of their octahedra.

**Figure 8:**
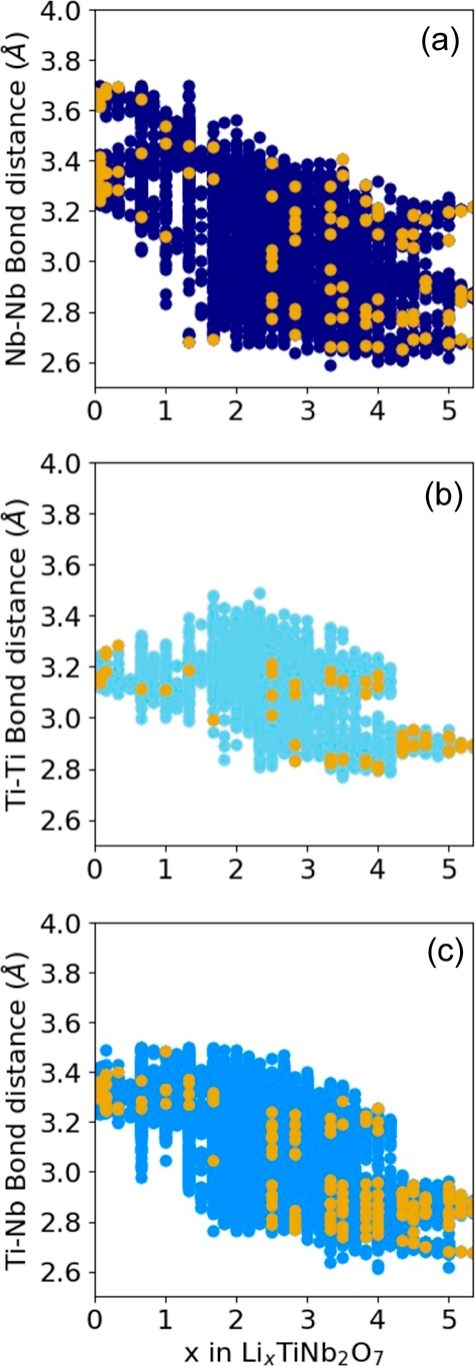
Bond lengths in Li_*x*_TiNb_2_O_7_ structures with formation energies within 50 meV/atom of the convex hull for edge-sharing (a) Nb–Nb pairs, (b) Ti–Ti pairs, and (c) Ti–Nb pairs. Gold points refer to the pair distances in ground state structures.

Figure 8 plots the edge-sharing metal–metal bond lengths as a function of the Li concentration collected from the 937 fully relaxed Li_*x*_TiNb_2_O_7_ structures. It is insightful to inspect the edge-sharing Nb–Nb, Nb–Ti, and Ti–Ti pair distances separately, as shown in Figure 8a–c, respectively. The nearest neighbor distances of edge-sharing metal–metal pairs in the ground-state configurations are shown in gold. The figures show a clear trend toward an overall contraction of the edge-sharing metal–metal bonds with increasing Li concentration. Especially notable is the abrupt contraction in a subset of the bond lengths that occurs between *x* = 1 and *x* = 2. In the lowest energy ground-state structures (gold points), this occurs first between Nb–Nb pairs, as is evident in Figure 8a, with the two gold points at *x* = 1.33 and *x* = 1.66, having values of approximately 2.7 Å. Edge-sharing Nb–Ti pairs (Figure 8c) also exhibit dimer formation, with a subset contracting to values close to 2.8 Å, but the contractions only set in appreciably after Nb–Nb pairs have started to contract. The contraction between edge-sharing Ti–Ti pairs (Figure 8b) is less pronounced and begins to occur only at higher Li concentrations.

**Figure 9:**
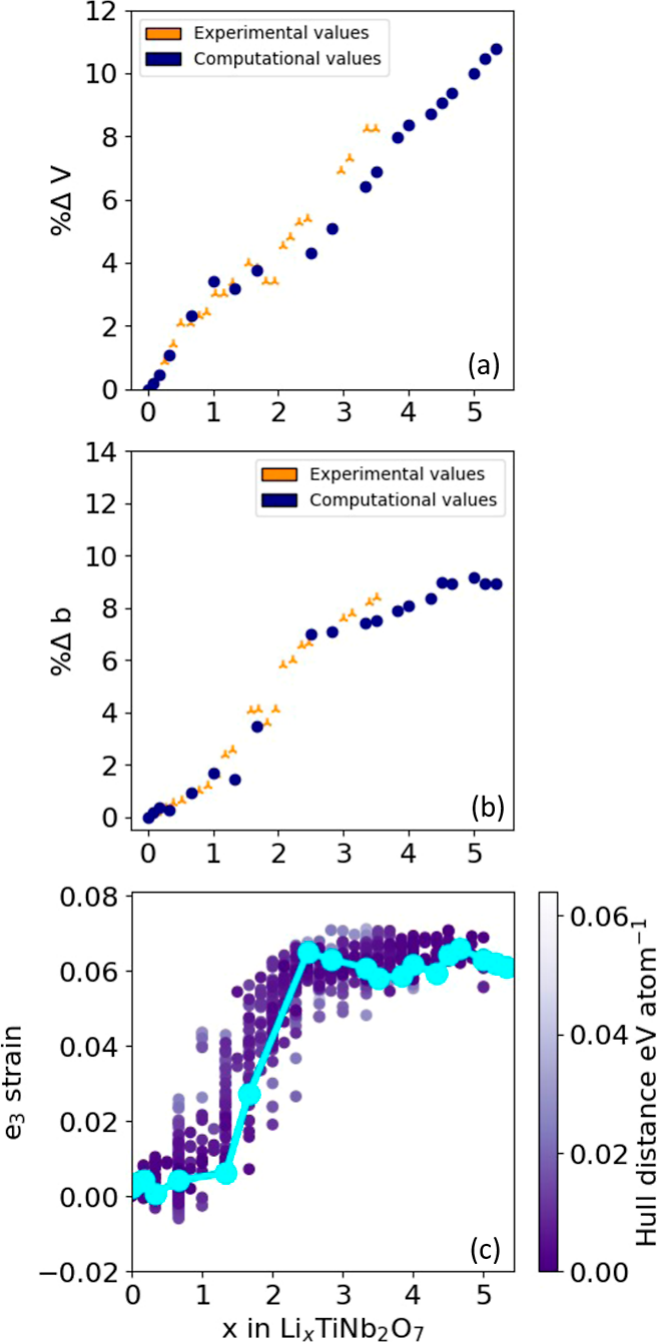
(a) Comparison of measured and calculated percent changes in the volume of the Li_*x*_TiNb_2_O_7_ unit cell. Points labeled as triangles were measured experimentally by Guo et al.^57^ Circles are for the ground-state structures of Li_*x*_TiNb_2_O_7_ as calculated with DFT-PBE. (b) Comparison of the percent change in the measured (triangles) and calculated (circles) *b* lattice parameter. (c) Calculated *e*_3_ strain relative to TiNb_2_O_7_ for 937 Li_*x*_TiNb_2_O_7_ structures.

The onset of metal–metal dimer formation leads to sizable dimensional changes in the host. Figure 9a collects the change in volumes of the relaxed ground-state structures of Li_*x*_TiNb_2_O_7_ relative to that of TiNb_2_O_7_ and compares them to the experimentally measured^57^ changes in volume. The agreement between the volume change, as calculated with DFT-PBE and the measured volume change, is very good. Figure 9b compares the change in the calculated lattice parameter *b* of the Li_*x*_TiNb_2_O_7_ host, which is parallel to the 3 × 3 blocks, for the ground-state structures to the corresponding experimental values.^57^ Here as well, the agreement is very good. The *b*-lattice parameter is predicted to increase abruptly between *x* = 1 and *x* = 2.5 and then levels off at higher Li concentrations. The same trend is observed experimentally.^57^

A useful metric of the dimensional changes of the host is the strain order parameter ,^19,58^ which measures tetragonal distortions along the 3 × 3 block axis of Li_*x*_TiNb_2_O_7_. The Cartesian strains, *E*_*xx*_, etc., appearing in the expression of the strain order parameter, *e*_3_, are defined with respect to a Cartesian coordinate system whose *ẑ* axis is parallel to the block length of the TiNb_2_O_7_ host structure. The Cartesian strains are calculated as Hencky strains^58^ relative to the dimensions of the fully relaxed TiNb_2_O_7_ host structure without any Li. Figure 9c collects the *e*_3_ strain for all 937 fully relaxed structures of Li_*x*_TiNb_2_O_7_. The *e*_3_ strains of the ground-state structures are connected by a light blue line. Similar to the variation in the *b*-lattice parameter in Figure 8b, the *e*_3_ strain order parameter shows a rapid increase in a narrow Li composition interval between *x* = 1 and *x* = 2.5. A positive value of *e*_3_ signifies an expansion along the block length and a contraction along the block waist. The abrupt increase in *e*_3_ and in the *b* lattice parameter between *x* = 1 and *x* = 2.5 can be attributed to the onset of metal–metal dimer formation, which results in a reduction of the distance between edge-sharing transition-metal cations and a straightening of the highly distorted octahedra of the pristine TiNb_2_O_7_ crystal structure.

**Figure 10:**
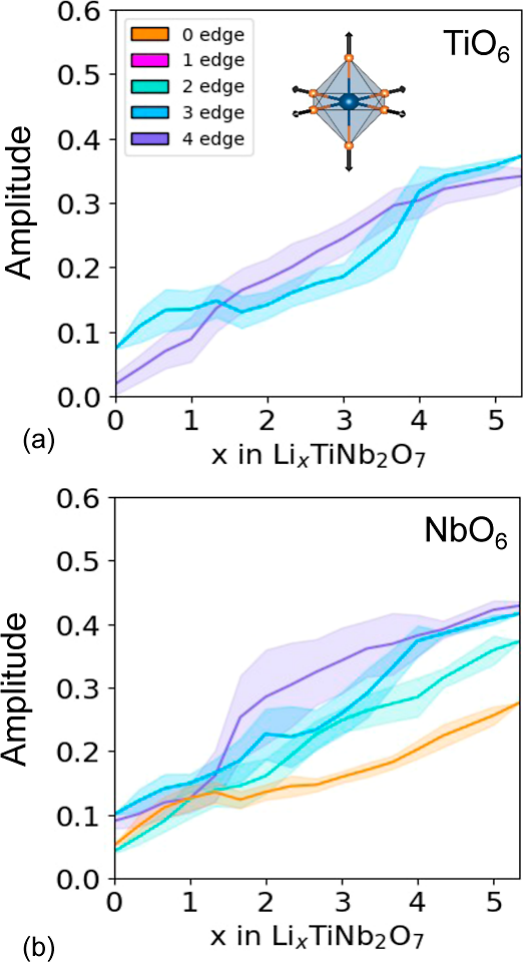
Amplitude of the octahedral breathing mode for (a) the TiO_6_ octahedra and (b) NbO_6_ octahedra as a function of lithium composition.

The degree with which the MO_6_ octahedra of TiNb_2_O_7_ distort upon Li insertion can be analyzed by projecting the ionic displacements of each octahedron on symmetry adapted collective displacements as described in the Supporting Information. Figure 10a,b, for example, shows the average amplitude of the symmetry preserving breathing mode of the TiO_6_ and NbO_6_ octahedra as a function of Li concentration. Also shown is one standard deviation spread around the average. The averages were collected from the 937 Li-vacancy orderings in Li_*x*_TiNb_2_O_7_ as relaxed with DFT-PBE. Figure 10 shows that the average volumes of the NbO_6_ and TiO_6_ octahedra increase steadily with the concentration of Li. Of particular interest in Figure 10b is the abrupt increase between *x* = 1 and *x* = 2 in the volume of the NbO_6_ octahedra that share four edges with neighboring octahedra (purple curve). This concentration interval coincides with the start of the metal–metal dimer redox mechanism, with the first pairs that form dimers involving Nb cations in octahedra that share four edges with neighboring octahedra. Figure 10b also shows that the Nb in the corner-sharing octahedron at the center of the 3 × 3 block (orange curve) expands between *x* = 0 and *x* ≈ 1 but then remains more or less constant until *x* ≈ 3. This is consistent with previous first-principles studies,^15,59,60^ which showed that the transition metal cations of the corner-sharing octahedra at the center of the blocks of Wadsley–Roth phases play an important role in the redox processes at dilute Li concentrations.

There are a total of 15 symmetry adapted collective displacement modes for a perfect MO_6_ reference octahedron. These naturally divide into six irreducible subspaces, as described in the Supporting Information. The breathing mode shown in the inset of Figure 10 forms a one-dimensional subspace. Another one is of dimension two and is spanned by the well-known first-order Jahn–Teller collective displacement modes. There are four additional irreducible subspaces, each of dimension three (see the Supporting Information for more details). Two of these are useful to analyze the distortion modes of Wadsley–Roth phases such as Li_*x*_TiNb_2_O_7_ as they measure the extent of second-order Jahn–Teller distortions and of the octahedral shape changes that accommodate the changes in the distance between edge-sharing cations.

**Figure 11:**
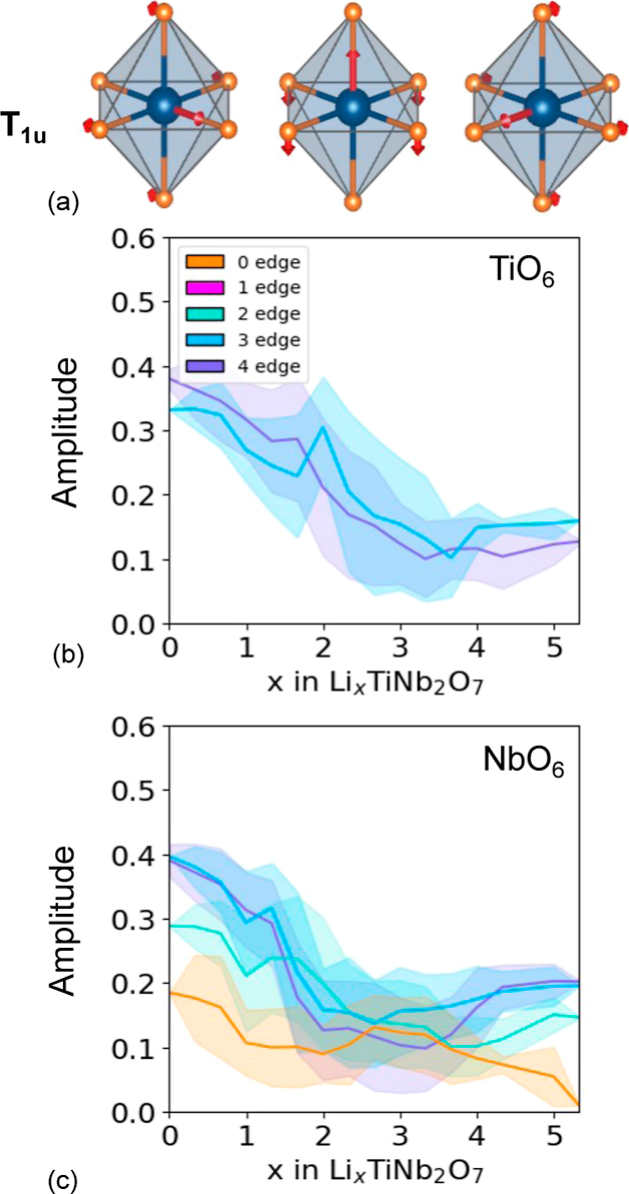
(a) Symmetry adapted collective displacement modes that characterize a second-order Jahn–Teller distortion of a d^0^ transition metal that is octahedrally coordinated by oxygen. The average amplitude of this type of displacement mode as a function of Li concentration for (b) TiO_6_ and (c) NbO_6_ octahedra. The amplitudes are calculated as a Euclidean distance within the space spanned by the three collective displacement modes of (a).

Figure 11a shows the three symmetry adapted collective displacement modes that characterize a second-order Jahn–Teller distortion of a d^0^ transition-metal coordinated by an octahedron of oxygen ions. The three collective displacement modes of Figure 11a each describe an off-centering of the transition metal along one of the Cartesian axes and form a basis on which to describe an arbitrary off-centering. A measure of the degree of off-centering is the Euclidean length of the three amplitudes of the collective distortion modes of Figure 11a, as described in the Supporting Information. Figure 11b,c shows the average Euclidean length of the off-centering distortion mode along with a one standard deviation spread for the TiO_6_ and NbO_6_ octahedra as a function of Li concentration. The averages were again taken over the 937 fully relaxed Li_*x*_TiNb_2_O_7_ structures. The large values at low Li concentrations indicate that the cations are displaced away from the center of their coordinating octahedra. The off-centering is more pronounced for the cations that share more edges with neighboring octahedra. The Nb of the corner-sharing octahedron at the center of the 3 × 3 block (orange curve in Figure 11c) exhibits the smallest degree of off-centering, which decreases abruptly around *x* ≈ 1. As the central Nb reduces its oxidation state from its starting value of Nb^5+^, its susceptibility to a second-order Jahn–Teller distortion is lowered, and the degree to which it is off-centered decreases. The other transition-metal cations, which are more off-centered at *x* = 0 than the central Nb due to the electrostatic repulsion with neighboring edge-sharing transition-metal cations, also become less off-centered with an increasing Li concentration. The decrease in the degree of off-centering occurs at slightly higher Li concentrations than that of the central Nb and coincides with the composition at which metal–metal dimers start to form. The formation of bonding states between neighboring transition-metal cations leads to metal–metal dimers, as described above, and an overall centering of the transition-metal cations within their octahedra.

**Figure 12:**
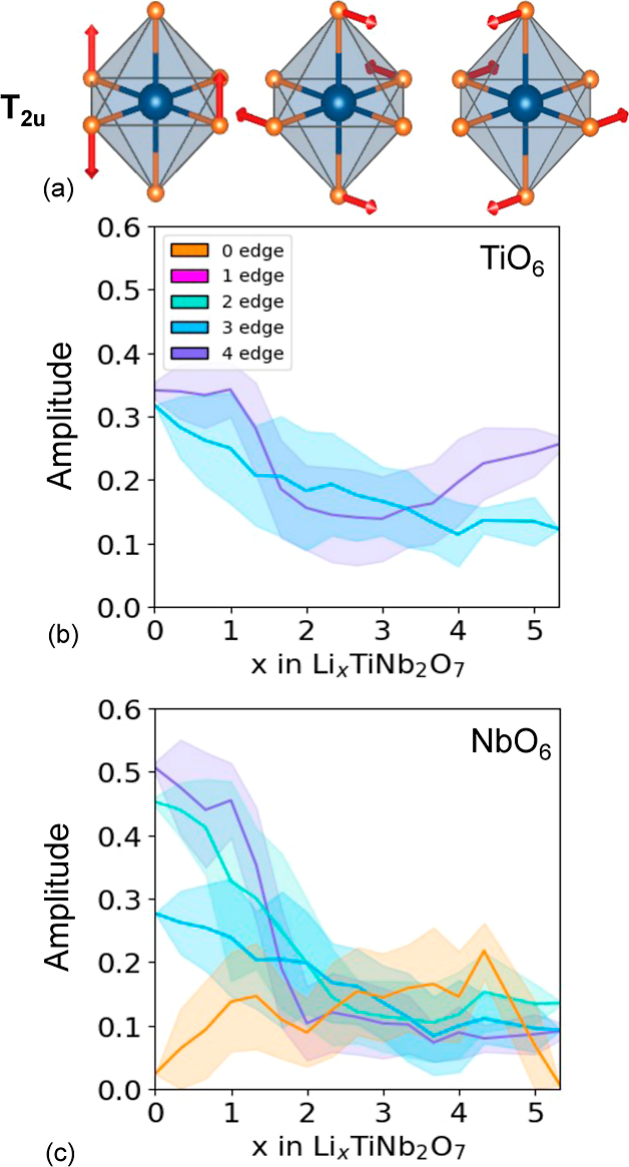
(a) Symmetry adapted the collective displacement modes of the oxygen octahedra that have large amplitudes in TiNb_2_O_7_. The average amplitude of this type of displacement mode as a function of Li concentration for (b) TiO_6_ and (c) NbO_6_ octahedra. The amplitudes are calculated as Euclidean distances within the space spanned by the three collective displacement modes of (a).

Figure 12a shows a second set of symmetry adapted collective displacement modes of MO_6_ octahedra whose amplitudes in Li_*x*_TiNb_2_O_7_ undergo large changes with Li concentrations. The three collective displacement modes of Figure 12a also form a basis to describe octahedral distortions that reside within a T_2*u*_ irreducible subspace, with each collective displacement involving four equatorial oxygen ions that distort perpendicular to their equatorial plane. These displacement modes measure the collateral distortions of the oxygen octahedra in response to the large relaxations that lead to an off-centering of edge-sharing transition-metal cations.^43^Figure 12b,c plots the average Euclidian distance of the amplitudes of the three orthogonal displacement modes for the TiO_6_ and NbO_6_ octahedra as a function of Li concentration. The octahedral distortions are large at dilute Li concentrations but decrease substantially over a small concentration interval between *x* = 1 and *x* = 2 upon the formation of metal–metal dimers. Upon formation of metal–metal dimers, the transition-metal cations move to the center of their octahedra, thereby allowing the oxygen ions to adopt positions that are closer to those of an ideal octahedron.

### 3.5 Finite Temperature Electrochemical Properties

Room-temperature electrochemical properties were calculated by combining cluster expansions with Monte Carlo simulations.^35,37^ A cluster expansion^61,62^ is a surrogate model that interpolates the energies of different Li-vacancy orderings as calculated with a computationally expensive first-principles method, such as DFT-PBE. The cluster expansion can then be used in Monte Carlo simulations to calculate the energies of microstates sampled in large unit cells according to the probability distribution of statistical mechanics.^37^ The cluster expansions used in this study were trained to the formation energies of the 937 Li-vacancy orderings in Li_*x*_TiNb_2_O_7_ shown in Figure 2a. A Bayesian approach was followed to enable uncertainty quantification of calculated thermodynamic properties due to numerical noise on the training data and cluster expansion truncation. Ten different cluster expansions were sampled from a Bayesian posterior probability distribution as described in Ober et al.^41^ Each cluster expansion was used in Monte Carlo simulations to calculate equilibrium voltage curves (related to the Li chemical potential according to the Nernst equation^37^) and equilibrium Li site occupancies as a function of the overall Li concentration.

**Figure 13:**
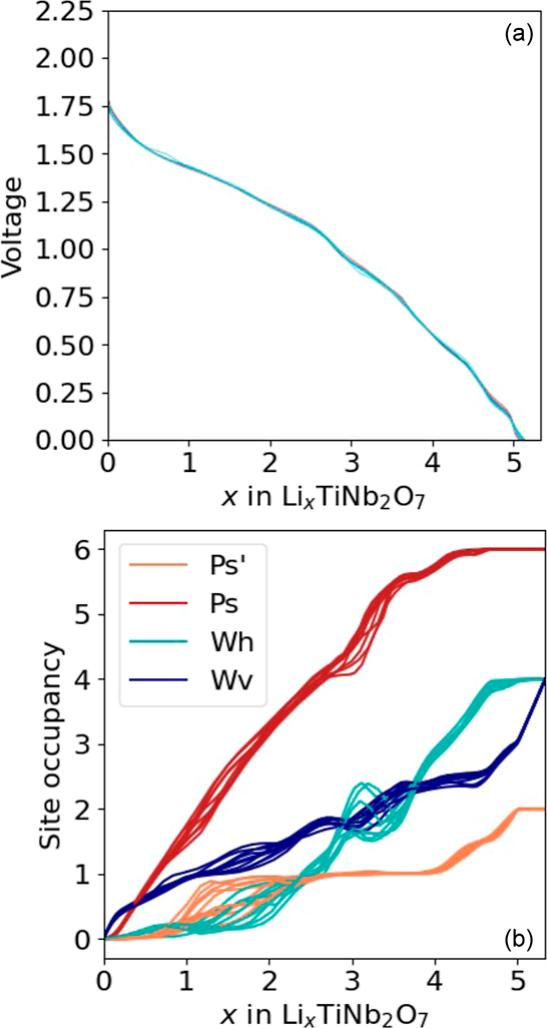
(a) Voltage profile at 300 K of Li_*x*_TiNb_2_O_7_ as calculated with Monte Carlo simulations applied to cluster expansions of the formation energy. The Ti–Nb ordering of TiNb_2_O_7_ is that of Figure 1. (b) Lithium site occupancy as a function of lithium concentration as calculated with Monte Carlo simulations applied to 10 different cluster expansions of the formation energy.

Figure 13a shows ten voltage curves as a function of Li concentration, each calculated with a different cluster expansion sampled from a Bayesian posterior distribution. The voltage curves were calculated with grand canonical Monte Carlo simulations, which generate the average Li concentration at each Li chemical potential and temperature. The smooth sloping voltage curves reflect a solid solution. The Monte Carlo simulations predict that the Li ions and vacancies are disordered at room temperature.

Similar to experimentally measured voltage profiles of Li_*x*_TiNb_2_O_7_,^12,25,63^ the calculated voltage curve exhibits an initial steep decrease between *x* = 0 and *x* ≈ 0.5, which is followed by a flatter concentration dependence between *x* ≈ 0.5 and *x* ≈ 2. Beyond *x* ≈ 2, the decrease in voltage with the Li concentration is again steeper and exhibits several weak steps. We note that the middle portion, while having a shallow slope, is not as flat as that exhibited by experimental curves.^11^

Figure 13b shows the Li site occupancy as a function of the Li concentration. Each Li site has ten curves, one for each cluster expansion sampled from the posterior distribution. It is clear in Figure 13b that differences in the predicted site occupancy as calculated with the different cluster expansions are small. The predicted trends in Figure 13b are consistent with those predicted at 0 K. Li primarily fills the pyramidal P_s_ sites, which steadily become enriched with Li until they saturate around *x* = 4. The vertical window sites, W_v_, also accommodate Li ions early on, but do not saturate until approximately *x* = 5. The horizontal window sites, W_h_, start filling only around *x* = 2. The pyramidal P_s_′ sites are overall the least favored sites and saturate only at the highest Li concentration.

**Figure 14:**
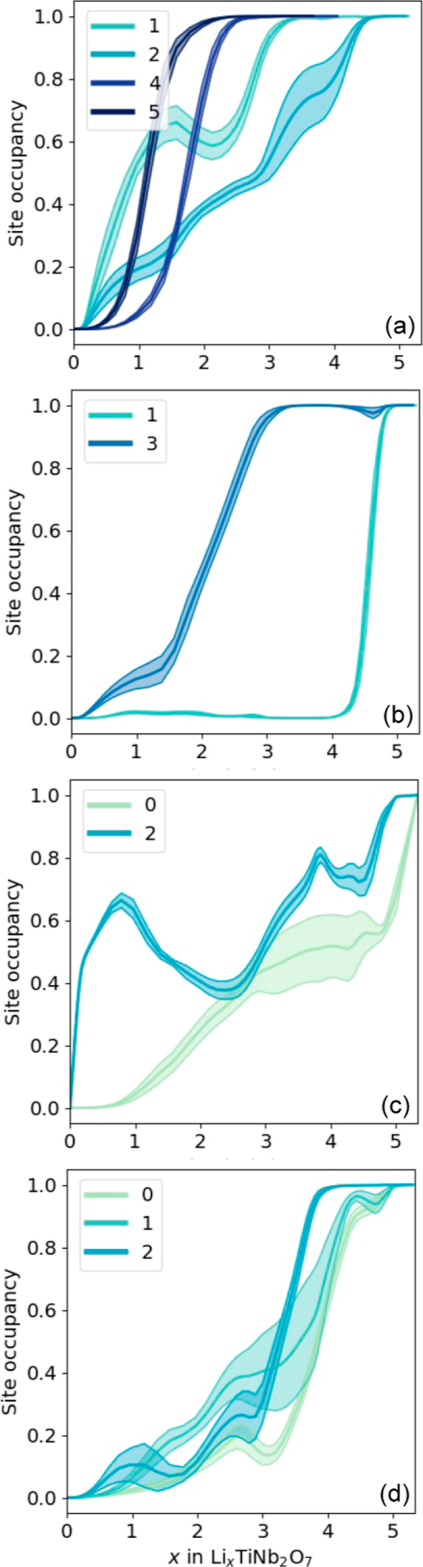
Li concentration of different sites coordinated by varying amounts of edge-sharing Ti for (a) P_s_, (b) P_s_′, (c) W_v_, and (d) W_h_ sites.

It is of interest to analyze the Li site occupancy based on the surrounding Ti concentration. This is shown in Figure 14. Each Li site is distinguished by the number of Ti cations that share an edge with the Li site. The darker blue curves and uncertainty bounds track the concentration of Li in sites that are surrounded by more Ti, as indicated in the insets, while the lighter green curves track the Li concentration in sites surrounded by more Nb. Overall, Figure 14 shows that Li tends to fill sites differently, depending on the amount of coordinating Ti. This is very starkly evident for the P_s_′ sites.

## 4 Discussion

Our first-principles study of the Li_*x*_TiNb_2_O_7_ Wadsley–Roth phase has shed light on the redox mechanisms accompanying the electrochemical lithiation of TiNb_2_O_7_. At least two redox mechanisms are identified based on an analysis of 937 fully relaxed Li_*x*_TiNb_2_O_7_ structures. At dilute Li concentrations, DFT-PBE calculations predict that electrons donated by Li reduce Ti and Nb more or less uniformly. The calculations predict some degree of spin polarization for *x* < 1. At higher Li concentrations, DFT-PBE calculations predict that the redox mechanism changes qualitatively, shifting from the filling of cation-centric t_2g_ orbitals to the filling of the bonding states that arise when the t_2g_ orbitals of edge-sharing transition-metal cations hybridize to form metal–metal dimers. The redox then occurs on extended molecular orbital-like states with enhanced charge density between pairs of edge-sharing transition-metal cations. The first metal–metal dimers to form involve Nb cations that occupy the sites with the highest number of edge-sharing neighbors. The large electrostatic interactions between highly oxidized edge-sharing neighbors increase the driving force to undergo redox at those sites in order to lower their formal oxidation state. Nb–Nb dimer formation is followed by Nb–Ti dimer formation with less pronounced activity predicted to occur between Ti–Ti pairs.

The dimer formation between edge-sharing transition metals has structural consequences. The distance between metal–metal pairs that host the electrons donated by Li in bonding states undergoes a contraction, which in turn induces a straightening of the oxygen octahedra surrounding the affected transition-metal cations. This leads to an elongation of the block length of the TiNb_2_O_7_ host that has macroscopic ramifications. The predicted variations in volume and *b* lattice parameter as a function of Li concentration are in good agreement with the experiment,^57^ indicating that DFT-PBE is capable of accurately describing the redox mechanisms in this material. The structural distortions of the host induced by metal–metal dimer formation affect not only the macroscopic dimensions of the crystal but also those of the interstitial Li sites. The window sites, for example, are highly distorted in the pristine TiNb_2_O_7_ structure and unfavorable for Li occupancy. Above *x* ≈ 2, however, when the MO_6_ octahedral distortions become less extreme, the window sites become more square planar and more favorable for Li occupancy.

The predicted variation in the magnetic moment of Li_*x*_TiNb_2_O_7_ as a function of Li concentration is also in very good agreement with the measurements of Griffith et al.^15^ DFT-PBE predicts some degree of spin polarization that leads to a net magnetic moment at low Li concentrations. The net magnetization is predicted to drop to negligible values, however, once the metal–metal dimer redox mechanism commences. The DFT-PBE calculations predict that the bonding states associated with the metal–metal dimers are filled by an equal number of spin up and spin down electrons and do not contribute to a net magnetic moment. Griffith et al.^15^ suggested that a Hubbard correction to DFT-PBE is necessary to describe the electronic structure of Li_*x*_TiNb_2_O_7_ at dilute Li concentrations. The analysis of a large number of Li-vacancy orderings at dilute concentrations in the current study, however, has shown that DFT-PBE without a Hubbard U correction is already capable of predicting the observed magnetic behavior as a function of Li concentration.

We expect that a redox mechanism involving the bonding states of metal–metal dimers is not restricted to Li_*x*_TiNb_2_O_7_, but it is common in other Wadsley–Roth phases as well. In fact, similar metal–metal bonding has been predicted to occur in Li_*x*_PNb_9_O_25_.^19^ However, because Li_*x*_PNb_9_O_25_ has only one type of transition metal and has a higher degree of cation ordering than Li_*x*_TiNb_2_O_7_, the metal–metal bonds are more extended, and the electronic states that emerge are more delocalized. Due to the presence of Ti and Nb disorder in Li_*x*_TiNb_2_O_7_, in contrast, the metal–metal dimer formation is more localized on individual edge-sharing pairs and therefore, more apparent as a mechanism of redox. Nb is known to form metal–metal bonds in other compounds, including NaNb_10_O_18_^64^ and NaNb_3_O_5_F,^65^ while metal–metal bonds involving Mo have been characterized in NaMoO_2_.^66^

The formation of metal–metal dimers to accommodate the charge donated by Li falls into a class of molecular-orbital like redox mechanisms that is increasingly being explored as an alternative to cation-centric redox mechanisms of conventional battery intercalation compounds.^32,67−69^ Other compounds exhibiting molecular-orbital redox mechanisms include Na_2_Mn_3_O_7_^32^ and Li_*x*_ScMo_3_O_8_.^68,69^ In Na_2_Mn_3_O_7_, redox has been predicted to occur on antibonding states distributed over an extended ring of π-bonded Mn and oxygen orbitals surrounding a cation vacancy.^32^ In Li_*x*_ScMo_3_O_8_, charge donated by Li is accommodated on molecular orbital-like states derived from Mo metal trimer clusters formed by the hybridization of t_2g_ orbitals.^68,69^

The redox mechanism described here for Li_*x*_TiNb_2_O_7_ induces structural changes due to the contraction in the distance between edge-sharing transition metal cations to form a favorable bonding state. This is similar to anion redox enabled by the formation of sulfur–sulfur S_2_^2^– dimers.^70−76^ More extreme redox mechanisms that require a change in the coordination environment include the Mn^4+^ → Mn^7+^ or the Cr^3+^ → Cr^6+^ redox couples that are accompanied by a migration from an octahedral site to an adjacent tetrahedral site.^77−79^ While significant structural changes due to redox processes are undesirable as they can lead to mechanical damage of the electrode material and hysteresis phenomena,^80,81^ the redox mechanism involving metal dimer formation is less extreme than coordination changing redox mechanisms. The crystallographic diversity of Wadsley–Roth phases, with widely varying numbers and distributions of edge-sharing octahedra, opens up opportunities to tailor the sequence of redox processes and their structural consequences.

As a final comment, we have discussed predictions for only one particular Ti–Nb ordering within the Wadsley–Roth structure of TiNb_2_O_7_. The Supporting Information shows that similar variations in the *b*-lattice parameter and volume occur for a different Ti–Nb ordering over the cation sites of the TiNb_2_O_7_ Wadsley–Roth structure. Many of the qualitative predictions of electrochemical properties for the Ti–Nb ground state ordering are also predicted for different Ti–Nb orderings, as summarized in the Supporting Information.

## 5 Conclusions

Our comprehensive first-principles study of Li insertion into the TiNb_2_O_7_ Wadsley–Roth phase has revealed an unusual redox mechanism that has important consequences for the structure of the host and Li site preferences as a function of Li concentration. While electrons fill t_2g_ orbitals centered on transition-metal cations of TiNb_2_O_7_ at dilute Li concentrations, they are accommodated by the bonding states of metal–metal dimers above *x* ≈ 1 in Li_*x*_TiNb_2_O_7_. The transition from cation centric redox to dimer bond redox centers results in significant structural changes of the host unit cell and oxygen octahedra coordinating transition-metal cations. The metal–metal dimer formation has consequences for the magnetic and electrochemical properties of the compound. The good agreement between predicted and experimentally measured magnetic moments and lattice parameter variations gives confidence in the validity of the predicted dimer redox mechanism. The insights of this work provide guidance as to how electrochemical properties can be tuned in early transition-metal oxide intercalation compounds by exploiting the rich structural and chemical diversity of Wadsley–Roth phases.^43^

## Supporting Information Available

Description of the symmetry adapted collective displacement modes of an octahedron and methods to calculate their amplitudes in fully relaxed structures; formation energies of TiNb_2_O_7_ orderings; Li-vacancy ordering ground states in Li_*x*_TiNb_2_O_7_; density of states and charge densities for multiple Li-vacancy ground states; zero-temperature predicted energies, voltage, and Li site occupancy profiles for a high symmetry TiNb_2_O_7_ ordering; and structural changes upon lithiation in a high symmetry TiNb_2_O_7_ ordering (PDF)
