# Supplementary material for: Redox Mechanisms, Structural Changes, and Electrochemistry of the Wadsley–Roth LixTiNb2O7 Electrode Material
Source: Chem Mater. 2023 Nov 14;35(22):9657–68. doi: 10.1021/acs.chemmater.3c02003 (PMC10687872; doi:10.1021/acs.chemmater.3c02003)
Supplement: Supplementary file 1 — cm3c02003_si_001.pdf [file cm3c02003_si_001.pdf]

Supporting Information

Redox mechanisms, structural changes, and electrochemistry of the Wadsley–Roth $Li_xTiNb_2O_7$ electrode material

Muna Saber,† Sesha Sai Behara,‡ and Anton Van der Ven*,‡

†*Department of Chemical Engineering, University of California, Santa Barbara, Santa Barbara, California 93106, United States*

‡*Materials Department, University of California, Santa Barbara, Santa Barbara, California 93106, United States*

E-mail: avdv@ucsb.edu

Analysis of octahedral deformations

The early transition metals that form Wadsley–Roth phases are usually in their maximum oxidation state. Transition metal cations such as Ti^{4+} and Nb^{5+}, have empty d-orbitals, which makes them susceptible to second-order Jahn-Teller distortions when octahedrally coordinated by oxygen. [1] There are also many edge-sharing TiO_6 and NbO_6 octahedra in $TiNb_2O_7$, which leads to strong repulsions between edge-sharing cations that result in large distortions of the octahedra.[2] It is of interest to quantify these distortions in $Li_xTiNb_2O_7$ as a function of Li concentration.

An MO_6 octahedron consists of six oxygen ions that coordinate a central transition metal, M. There are therefore $7 \times 3 = 21$ displacement degrees of freedom that characterize the position,

orientation and deformation state of an octahedron. Three degrees of freedom describe a rigid translation of the octahedron while an additional three degrees of freedom describe a rigid rotation of the octahedron. The remaining $21 - 6 = 15$ degrees of freedom describe distortions of the octahedron relative to an ideal reference octahedron. It is convenient to analyze these distortions in terms of symmetry adapted collective displacements of the atoms of an ideal octahedron, as these decompose a general distortion into a linear superposition of different group/subgroup symmetry breaking collective distortions.[3–6] As an example, Figure S1 shows 15 symmetry adapted collective displacement modes of an octahedron divided into six irreducible subspaces according to the cubic point group of an octahedron.

Before the distortions of a cluster can be decomposed into a superposition of symmetry adapted collective displacements, it is first necessary to remove the rigid translation and rotation of the cluster relative to an ideal reference cluster.[3, 5, 7] The position coordinates of the atoms of an N-site cluster can be collected into a $3 \times N$ matrix $\mathbf{R} = [\vec{r}_1, \ldots, \vec{r}_N]$, where each $\vec{r}_i$ is a column vector of the Cartesian coordinates of the atom at site i. In a similar way, the coordinates of the atoms of an ideal, high symmetry N-site reference cluster can be collected in the matrix $\mathbf{R_0} = [\vec{r}^{\,0}_1, \ldots, \vec{r}^{\,0}_N]$. Any rigid translation can be removed by placing the geometric center of mass of the distorted and ideal cluster at the origin of the Cartesian coordinate system. The coordinates of the distorted cluster can then be related to those of the ideal reference cluster according to

$$\mathbf{R} = \mathbf{U}(\mathbf{R}_0 + \mathbf{D}) \tag{1}$$

where $\mathbf{U}$ is a 3×3 rotation matrix and $\mathbf{D}$ is a $3 \times N$ matrix whose columns are displacement vectors, i.e. $\mathbf{D}$=$[\vec{d}_1, \ldots, \vec{d}_N]$. Equation 1 states that the distorted cluster is obtained by first displacing each atom of the ideal reference cluster at position $\vec{r}^{\,0}_i$ by the vector $\vec{d}_i$ and then rotating the distorted cluster with the rotation matrix $\mathbf{U}$ to generate the coordinate $\vec{r}_i$. A rotation matrix is unitary such that $\mathbf{U}^{-1} = \mathbf{U}^{\mathsf{T}}$.

The order of the atoms in the distorted polyhedron relative to the reference polyhedron as they appear in the matrices $\mathbf{R}$ and $\mathbf{R_0}$ determines the rotation matrix $\mathbf{U}$ and displacement vectors

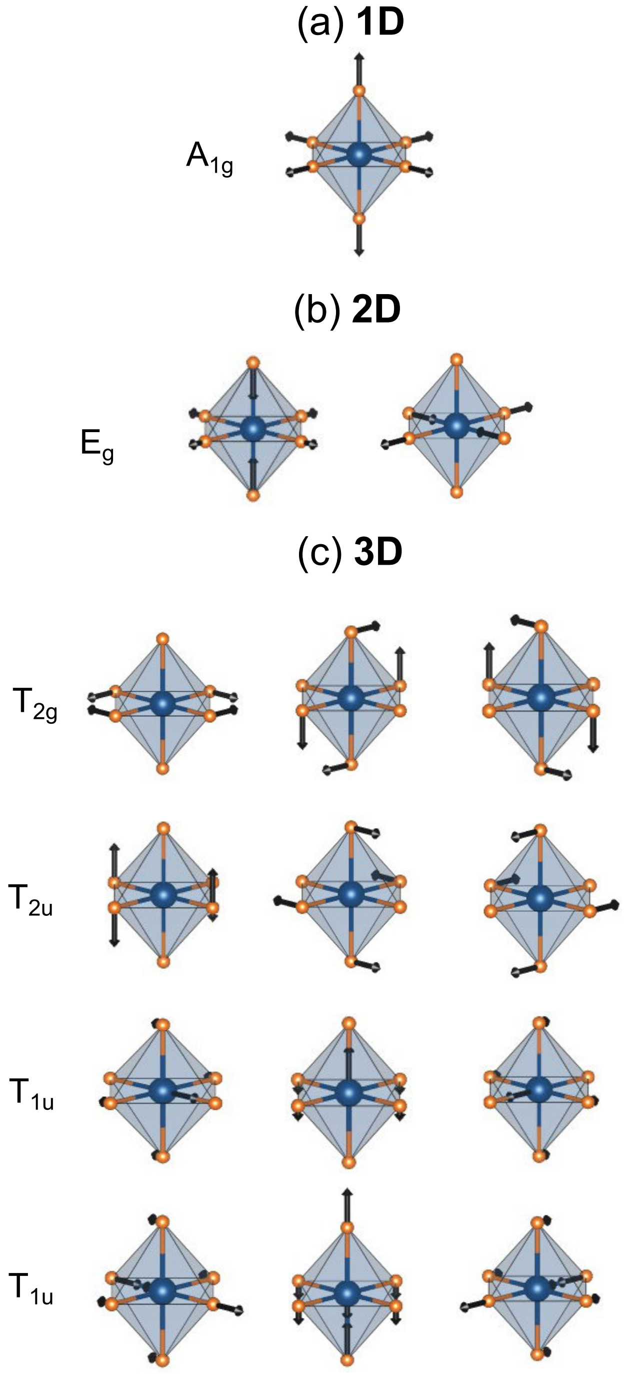

Figure S1: Symmetry adapted collective displacements for an octahedron with seven atoms including (a) one one-dimensional irrep corresponding to volumetric distortions of point point group A_{1g}, (b) one two-dimensional irrep corresponding to tetragonal distortions of point group E_g, (c) four three-dimensional irreps corresponding to two T_{1u} distortions, one T_{2g} distortion and one T_{2u} distortion.

$\mathbf{D}$. An assignment of atoms from the distorted cluster to those of the reference cluster must be chosen to minimize the displacements at each site. The Hungarian algorithm can be used to determine the optimal assignment of atomic positions of the distorted polyhedron.[8] This algorithm is applied before the optimal rotation matrix $\mathbf{U}$ is calculated.

The rotation matrix $\mathbf{U}$ is chosen such that it minimizes the sum of the square displacements between the distorted and the reference cluster

$$\sum_{i=1}^{N} |\vec{d_i}|^2 = \sum_{i=1}^{N} |\mathbf{U}^{\mathsf{T}}\vec{r_i} - \vec{r_i^0}|^2 \tag{2}$$

where the $\vec{d_i}$ are the columns of $\mathbf{D}$ and where $|\vec{d_i}|$ is the length of the vector $\vec{d_i}$. The Kabsch algorithm[9–11] can be used to find the rotation matrix $\mathbf{U}$ that minimizes Eq. 2. The displacements $\mathbf{D}$, as defined by Eq. 1 using the optimal rotation matrix $\mathbf{U}$ that minimizes Eq. 2, then describes the deformation state of the distorted cluster relative to an ideal high symmetry cluster. These displacements are to be decomposed into a superposition of symmetry adapted collective displacements.

To decompose the deformation state of a cluster in terms of symmetry adapted collective displacements, it is convenient to unroll the $3 \times N$ matrix of displacements $\mathbf{D}$ into a $3N$ column vector, $\vec{D}^{\mathsf{T}} = [\vec{d_1}^{\mathsf{T}}, \ldots, \vec{d_N}^{\mathsf{T}}]$. The symmetry adapted collective displacements relative to the high symmetry reference cluster can likewise be assembled in column vectors $\vec{q_j}$ of length $3N$. There are a total of $3N-6$ symmetry adapted collective displacements that describe deformations and these are orthogonal to the 3 collective displacements that describe a rigid translation of the cluster and the 3 collective displacements that describe infinitesimal rotations of the reference cluster. The displacement field of the distorted cluster can therefore be expressed as a linear superposition of the $3N-6$ symmetry adapted collective modes $\vec{q_j}$ according to

$$\vec{D} = \sum_{j=1}^{3N-6} \alpha_j \vec{q_j} \tag{3}$$

where the coefficients α_j are the amplitude of each symmetry adapted collective displacement. In our analysis of the octahedral distortions in Wadsley–Roth phases, we project the displacements as collected in the 21-dimensional column vector $\vec{D}$ onto the 15 symmetry adapted collective modes, $\vec{q}_1, \ldots, \vec{q}_{15}$, shown in Figure S1. The symmetry adapted collective modes can be generated with well-established group theoretical techniques.[12] Those shown in Figure S1 were generated algorithmically as described in Thomas and Van der Ven.[4]

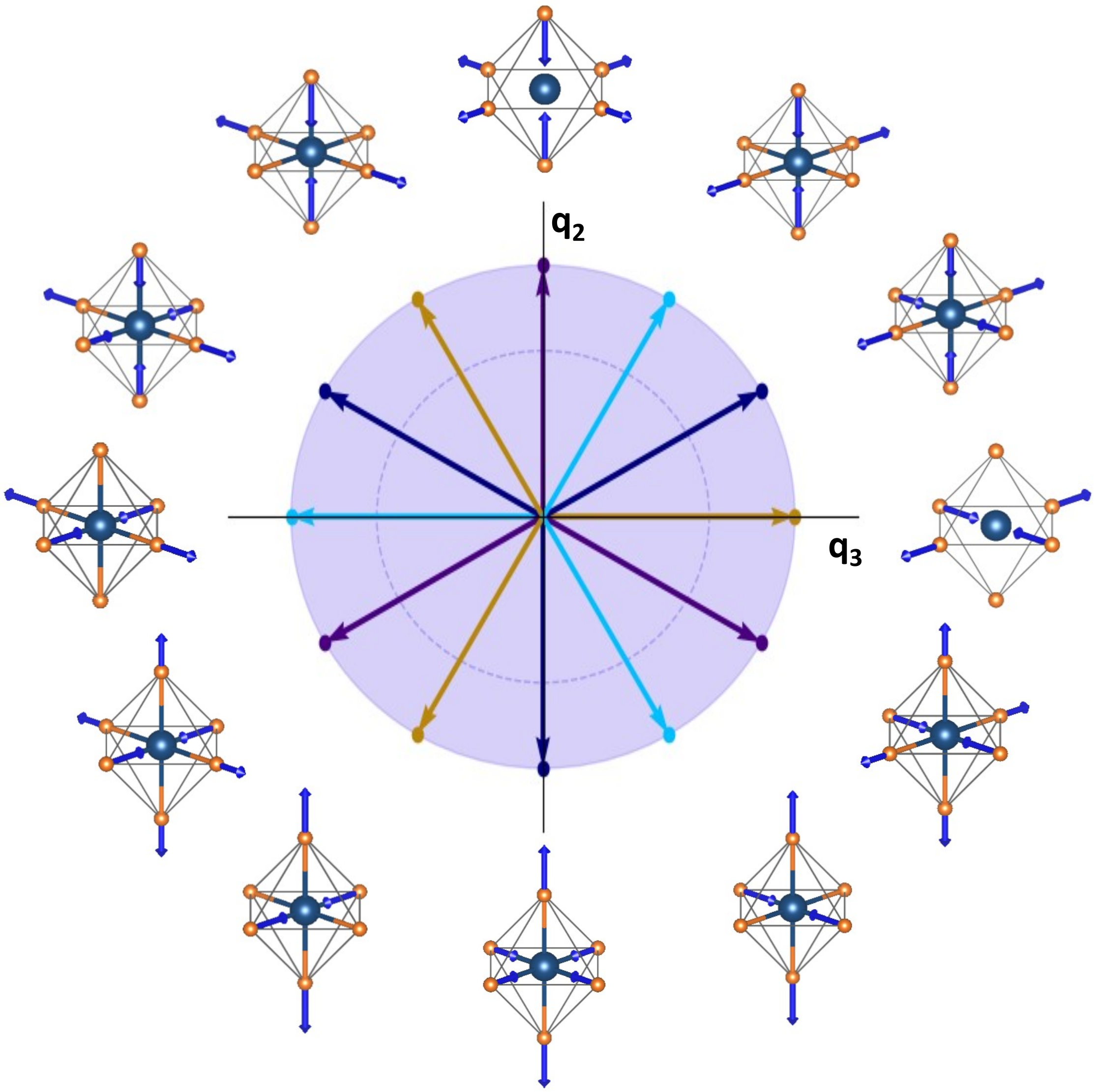

Figure S2: Representation of the norm of the two dimensional $\Gamma_2(E_g)$ irrep.

The symmetry adapted collective displacements, $\vec{q}_1, \ldots, \vec{q}_{15}$, fall into different irreducible subspaces. The first irreducible subspace (irrep) for the octahedron ($\vec{q}_1$), shown in Figure S1(a), spans

a one-dimensional subspace of the 21-dimensional space of displacement degrees of freedom and describes the symmetry preserving breathing mode of an octahedron. The second irrep of the octahedron ($\vec{q}_2$, $\vec{q}_3$), (Figure S1(b)), spans a two-dimensional subspace and describes tetragonal and orthorhombic distortions of the cubic reference octahedron. These symmetry adapted collective displacements describe the well-known first-order Jahn-Teller distortions of an octahedrally coordinated transition metal with degenerate e_g valence electron configuration.[13–15] All symmetrically equivalent distortions reside within the same irrep. This is illustrated in Figure S2 for the E_g irrep of the octahedron spanned by $\vec{q}_2$ and $\vec{q}_3$. The coordinates in this subspace are the amplitudes α_j appearing in Eq. 3. The points and lines having the same color represent symmetrically equivalent distortions of the octahedron according to the cubic point group of the undistorted reference octahedron. There are an additional four irreps of symmetry adapted collective modes, each of dimension 3, which are collected in Figure S1(c).

The collection of amplitudes, α_j, of a distortion within a 2-dimensional or 3-dimensional irreducible subspace forms a vector in that space. While the full vector of amplitudes within any given irrep is necessary to construct the precise symmetry breaking distortion allowed by that irrep, it is often sufficient to simply track whether a particular irrep is prominent in a distorted cluster. This can be assessed by simply calculating the length of the amplitude vector within each irrep.[16] The Euclidean norm within each irrep takes the form

$$\gamma_i = \sqrt{\sum_{j \in \Gamma_i} \alpha_j^2} \tag{4}$$

where γ_i is the length of the amplitudes in irrep Γ_i and where the sum extends over all the amplitudes α_j within the irrep (i.e. $j \in \Gamma_i$).

Transition metal ordering in $TiNb_2O_7$

The $TiNb_2O_7$ compound adopts the $E_1[3 \times 3]$ Wadsley–Roth structure (Figure S3). There are five symmetrically inequivalent sites that may accommodate titanium. The M1 and M2 octahedra share four edges with neighboring octahedra. The M3 octahedral site shares edges with 3 octahedra. The M4 octahedral site is central in the 3×3 block and is purely corner-sharing with other octahedral sites. The M5 octahedral site shares two octahedral edges. The octahedral site naming scheme was introduced by Dreele et al. [17]

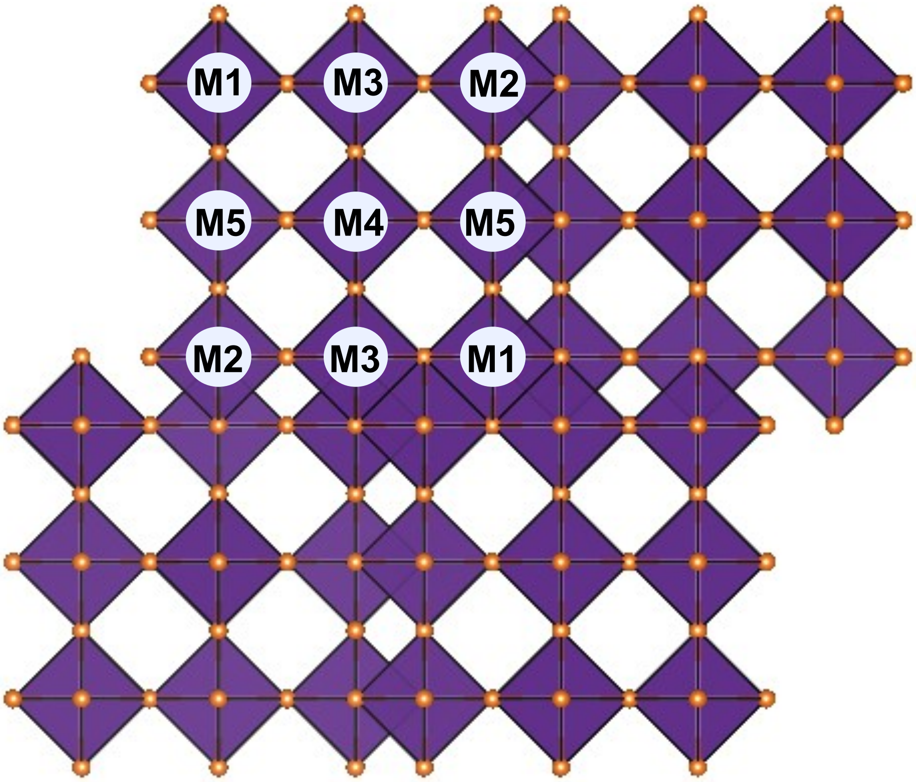

Figure S3: The $E_1[3 \times 3]$ Wadsely–Roth structure of $TiNb_2O_7$. There are 5 symmetrically inequivalent transition metal sites that can be occupied by Ti and Nb cations. These are referred to as M1,. . . , M5. The numbering scheme was introduced by Dreele et al. [17] Adapted from Saber and Reynolds et al. [2]

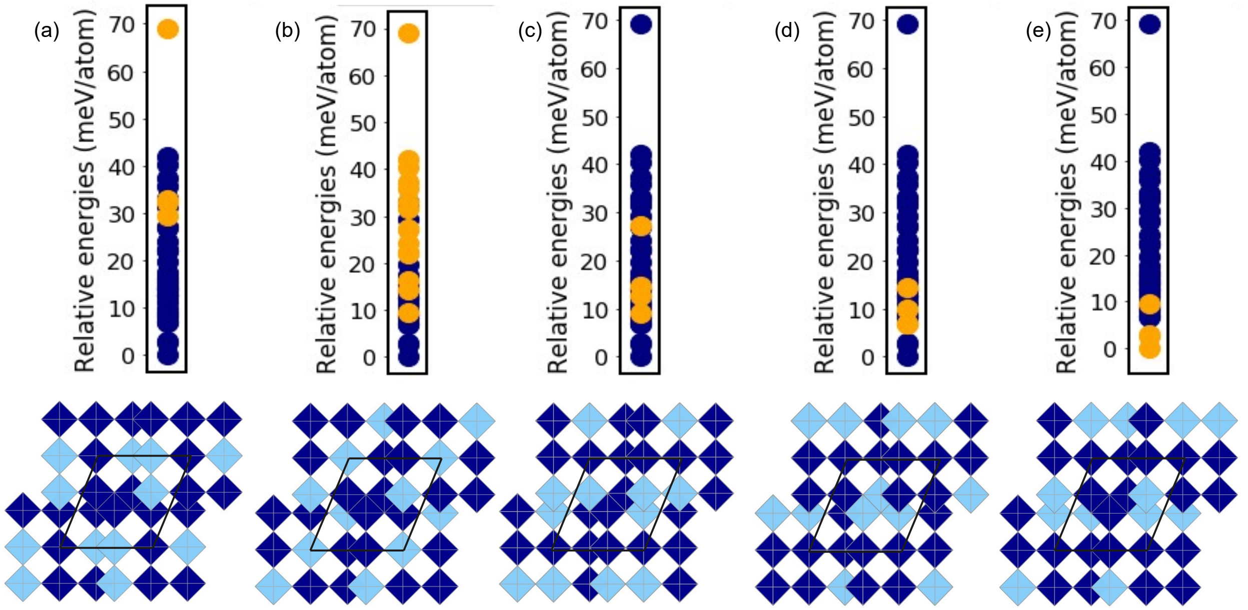

Figure S4: The energies of 44 different Ti-Nb orderings over the cation sites of $TiNb_2O_7$. Each column highlights in gold the energies of different orderings having one of the transition metal sites exclusively filled by Ti. The gold points in (a) correspond to all Ti-Nb orderings in $TiNb_2O_7$ that have all M5 sites filled by Ti. The ordered arrangement in the $E_1[3 \times 3]$ structure corresponding to the lowest energy ordering in which all M5 sites are filled by Ti is shown below the energy plot. Light blue are octahedral sites filled by Ti while dark blue are octahedral sites filled by Nb. Similar plots are shown for orderings with all M4 sites filled by Ti (b), all M3 sites filled by Ti (c), all M1 sites filled by Ti (d) and all M2 sites filled by Ti (e). The zero on the energy axis is referenced to the lowest energy Ti-Nb ordering within the $TiNb_2O_7$ structure.

Li-vacancy ground state orderings in $Li_xTiNb_2O_7$

The ground state Li-vacancy orderings in the $TiNb_2O_7$ host with the lowest energy Ti-Nb ordering are shown below.

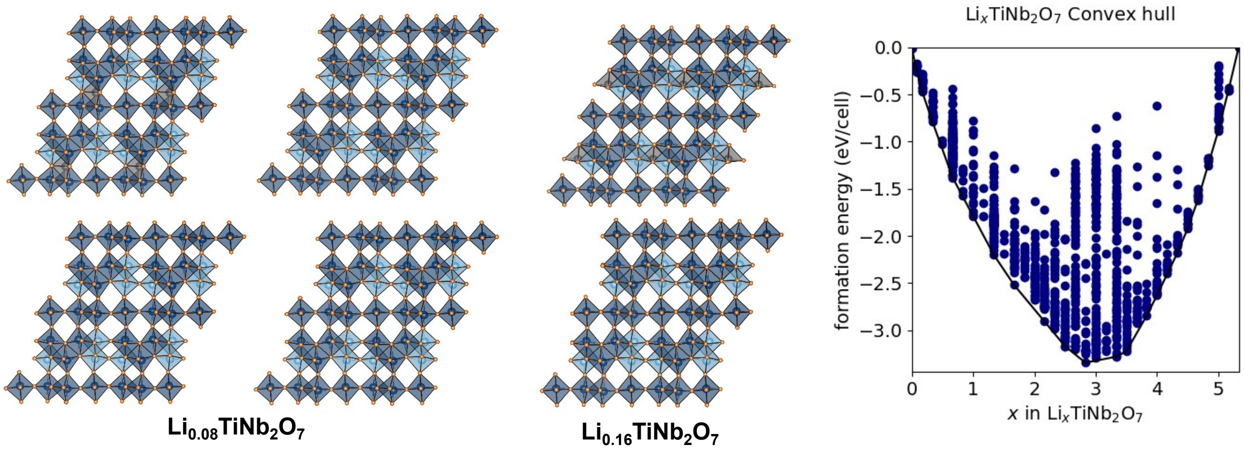

Figure S5: Ground state lithium-vacancy orderings at $x = 0.08$ and $x = 0.16$. Each image is a slice along the b lattice vector of the $TiNb_2O_7$ host structure. There are four images for the $x = 0.08$ ground state since the Li ordering in this ground state forms a super lattice with a four fold increase in the b axis. The ground state at $x = 0.16$ requires a doubling of the b lattice vector. Light blue atoms are Ti, dark blue atoms are Nb and grey atoms are Li.

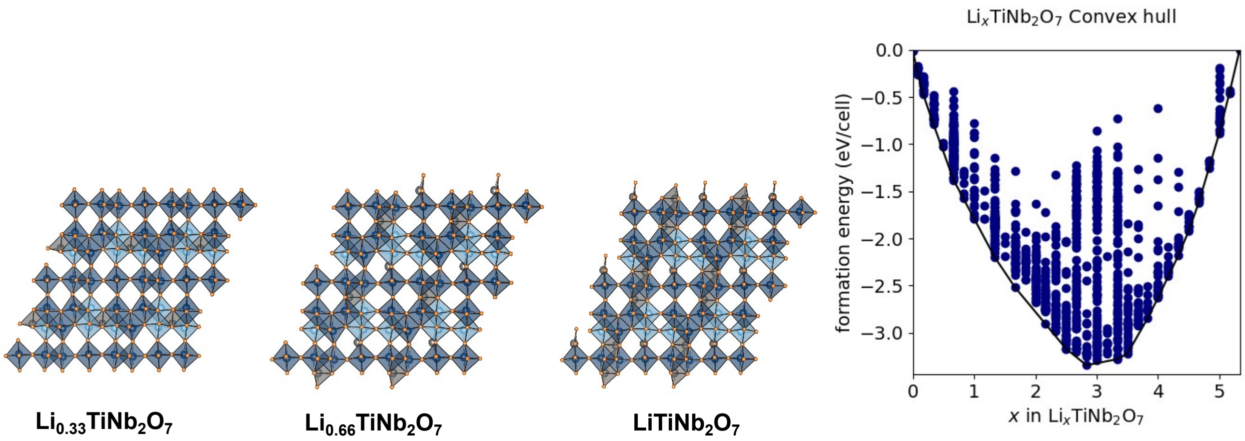

Figure S6: Ground state lithium-vacancy orderings at $x = 0.33$, $x = 0.66$, and $x = 1$.

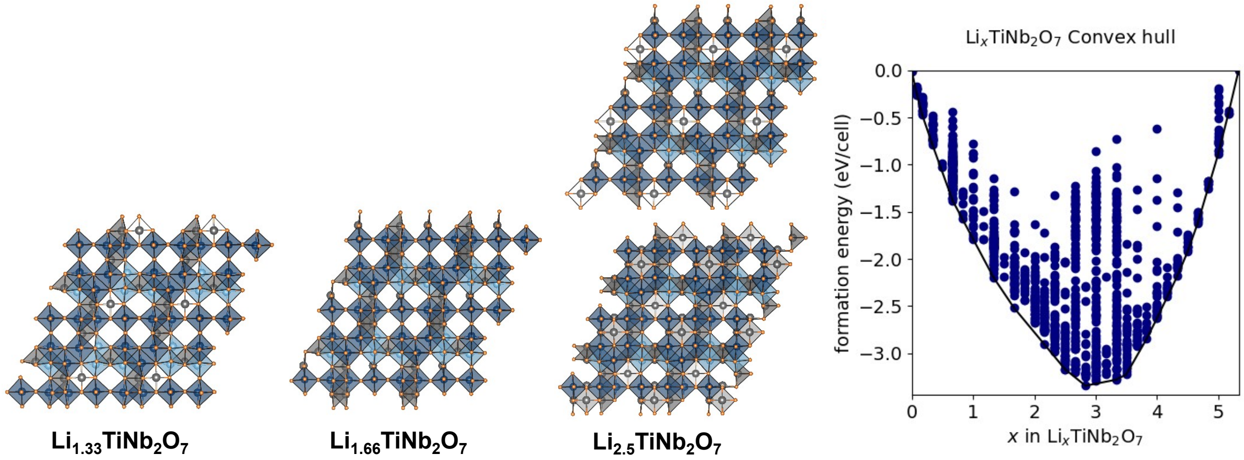

Figure S7: Ground state lithium-vacancy orderings at $x = 1.33$, $x = 1.66$, and $x = 2.5$.

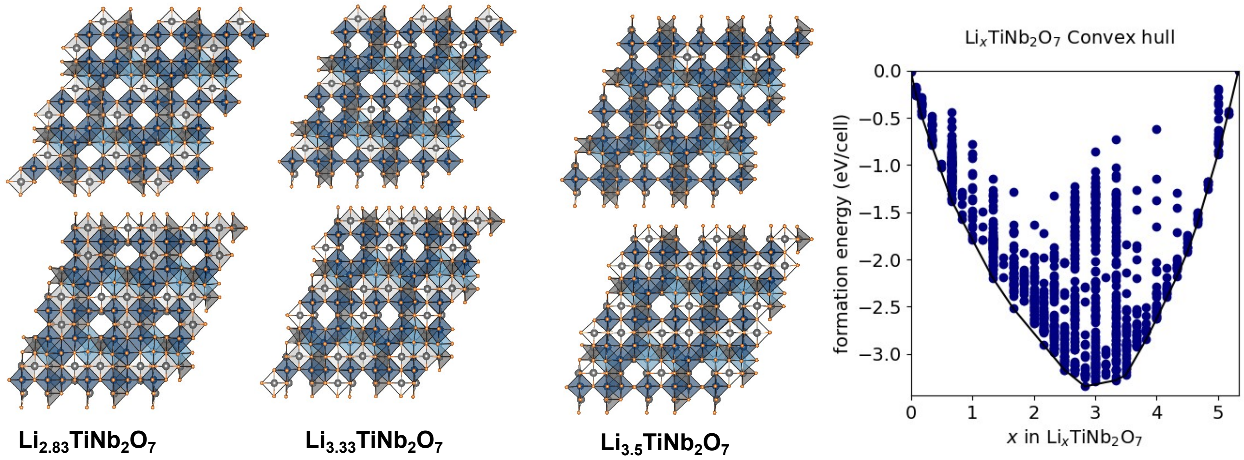

Figure S8: Ground state lithium-vacancy orderings at $x = 2.83$, $x = 3.33$, and $x = 3.5$.

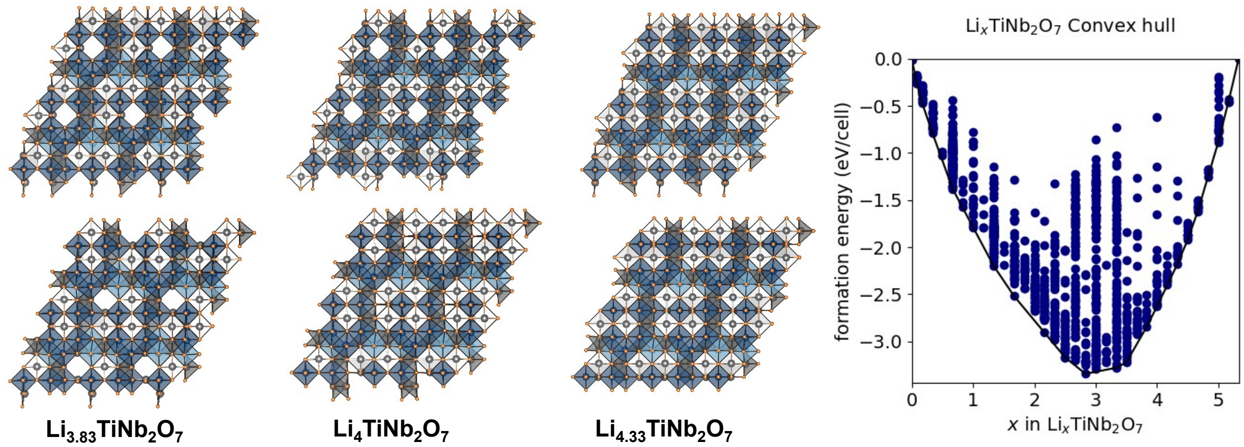

Figure S9: Ground state lithium-vacancy orderings at $x = 3.83$, $x = 4$, and $x = 4.33$.

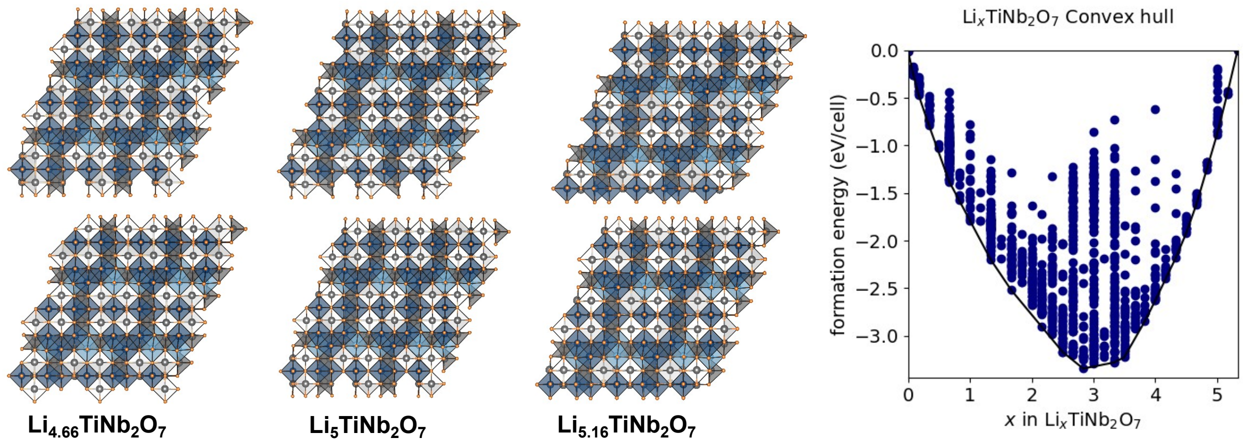

Figure S10: Ground state lithium-vacancy orderings at $x = 4.66$, $x = 5$, and $x = 5.16$.

Electronic density of states

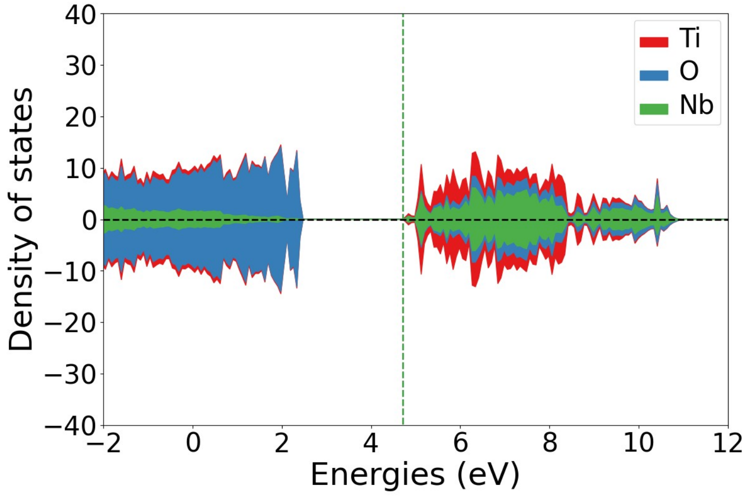

Figure S11: Density of states for $TiNb_2O_7$.

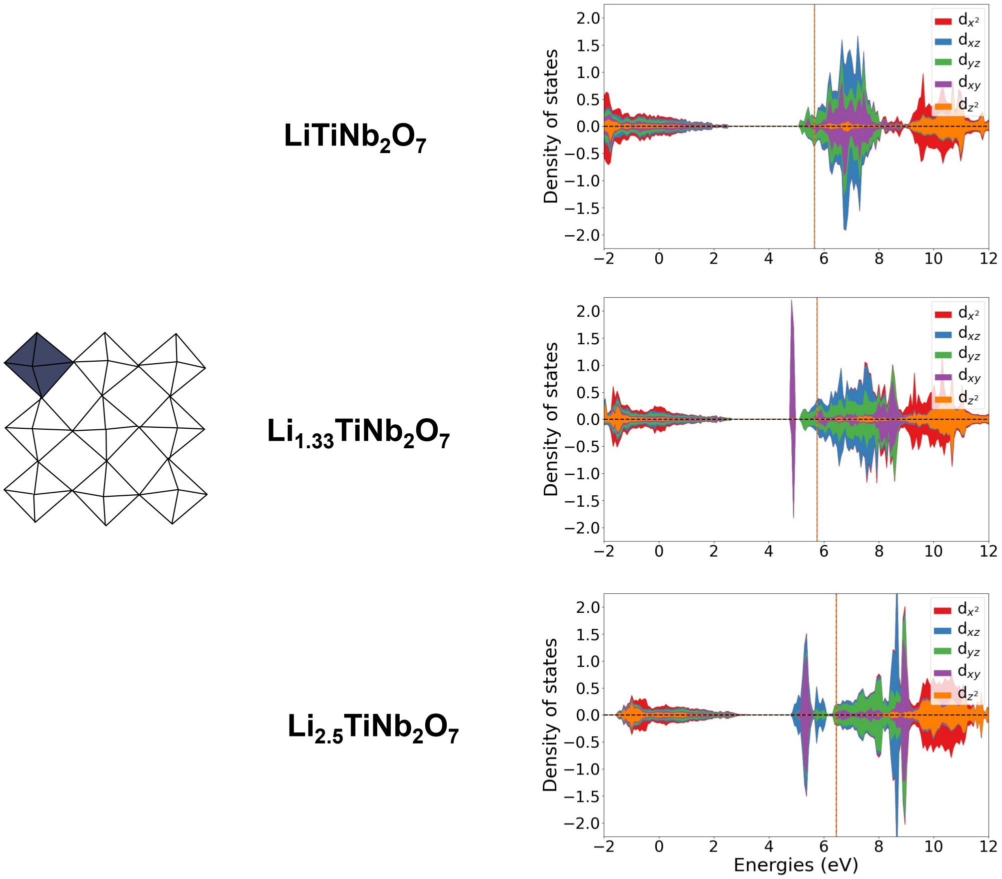

Figure S12: Projected density of states for the M1 Nb Site for $x = 1$, $x = 1.33$, and $x = 2.5$ in $Li_xTiNb_2O_7$.

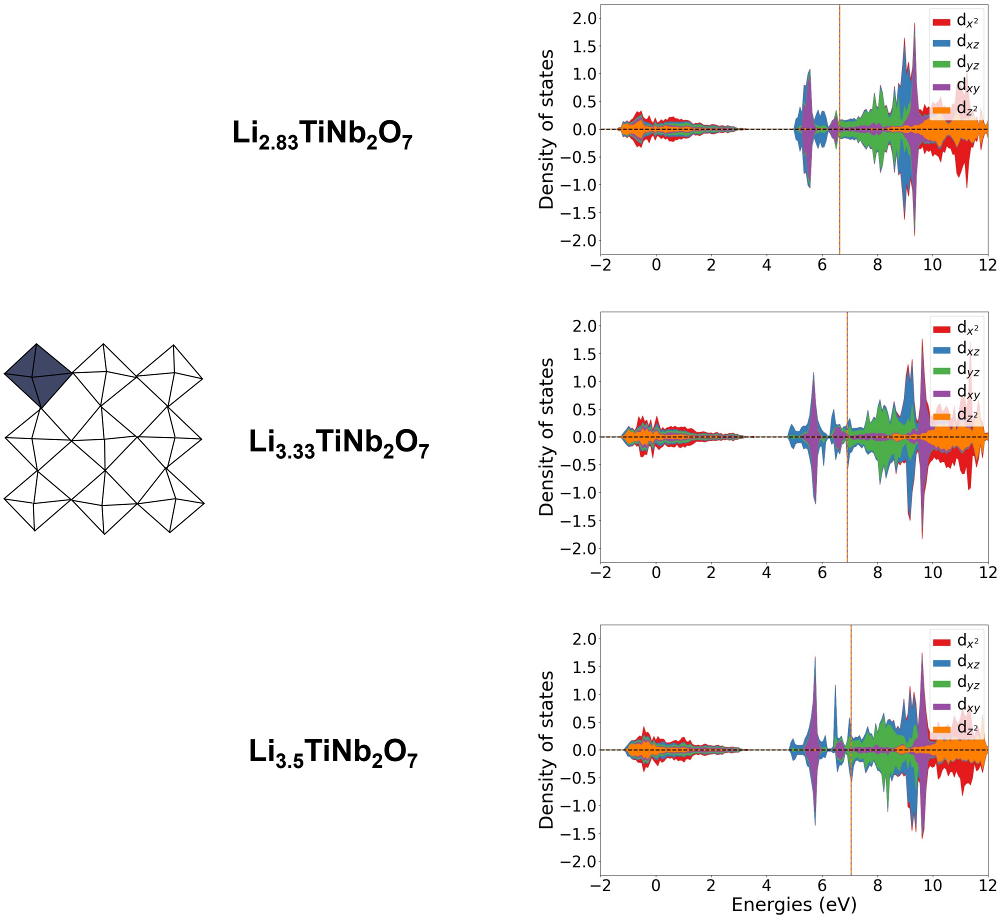

Figure S13: Projected density of states for the M1 Nb Site for $x = 2.83$, $x = 3.33$, and $x = 3.5$ in $Li_xTiNb_2O_7$.

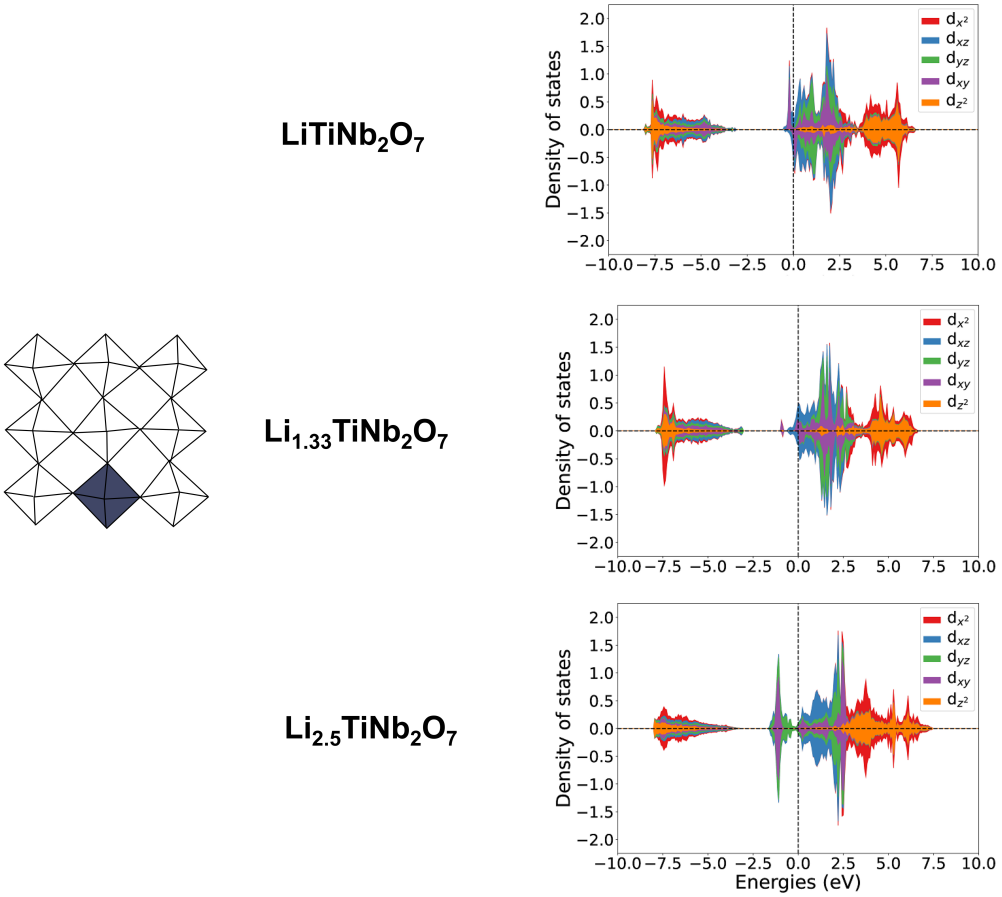

Figure S14: Projected density of states for the M3 Nb Site for $x = 1$, $x = 1.33$, and $x = 2.5$ in $Li_xTiNb_2O_7$.

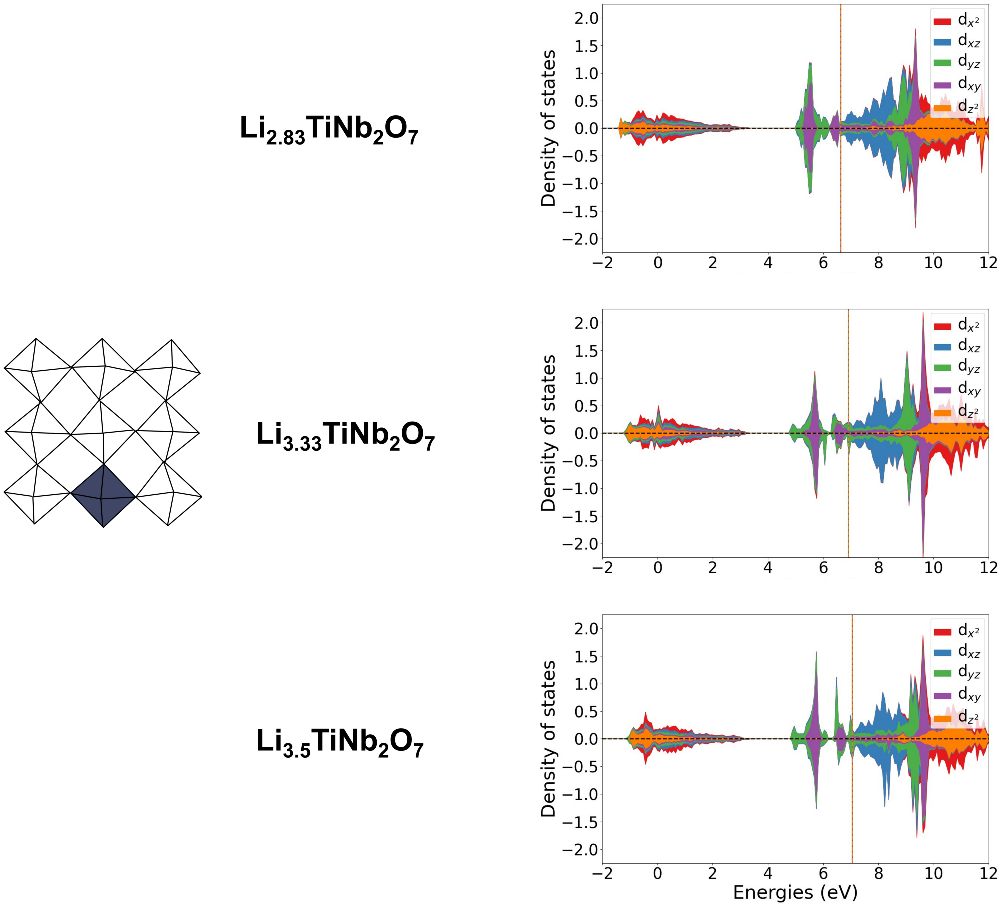

Figure S15: Projected density of states for the M3 Nb Site for $x = 2.83$, $x = 3.33$, and $x = 3.5$ in $Li_xTiNb_2O_7$.

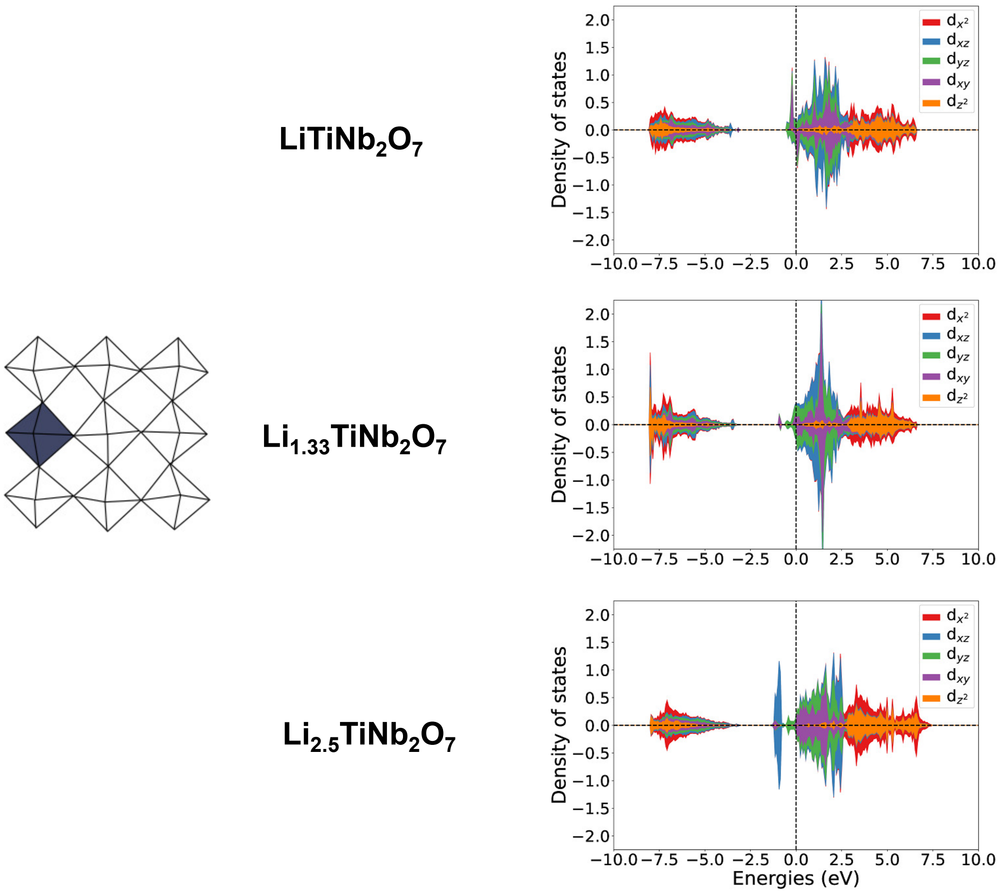

Figure S16: Projected density of states for the M5 Nb Site for $x = 1$, $x = 1.33$, and $x = 2.5$ in $Li_xTiNb_2O_7$.

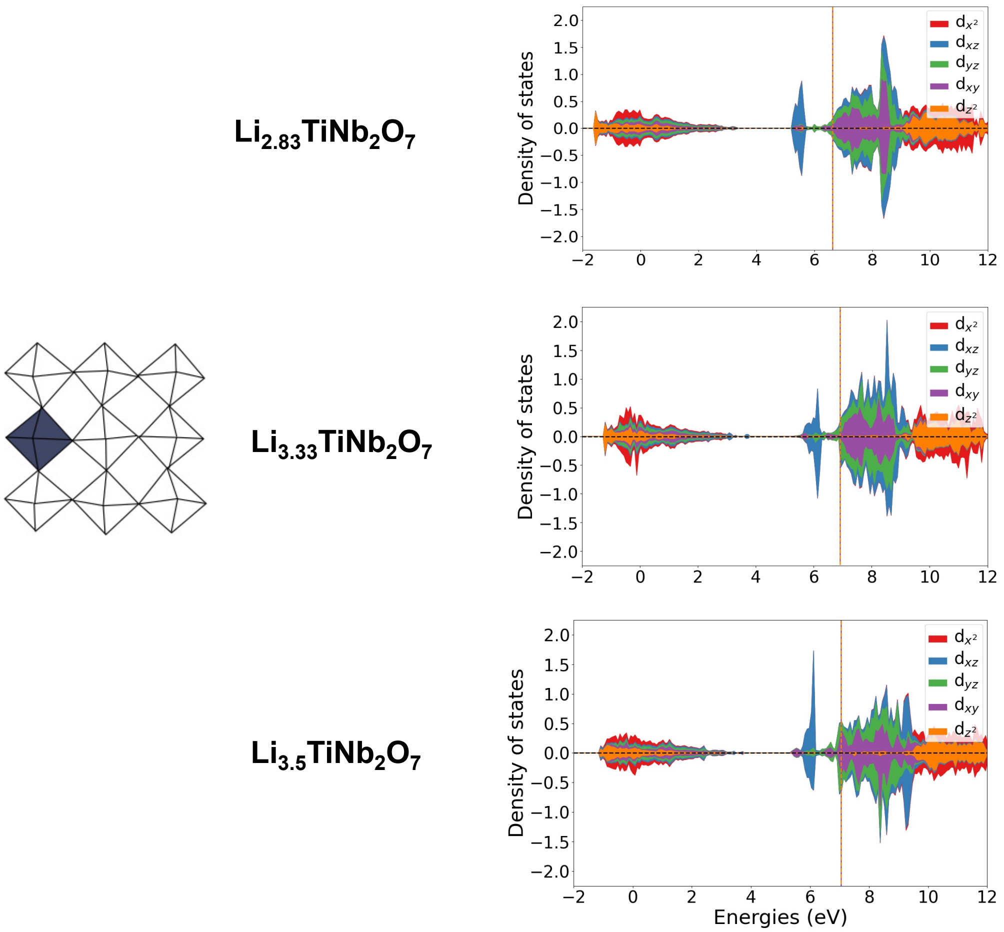

Figure S17: Projected density of states for the M5 Nb Site for $x = 2.83$, $x = 3.33$, and $x = 3.5$ in $Li_xTiNb_2O_7$.

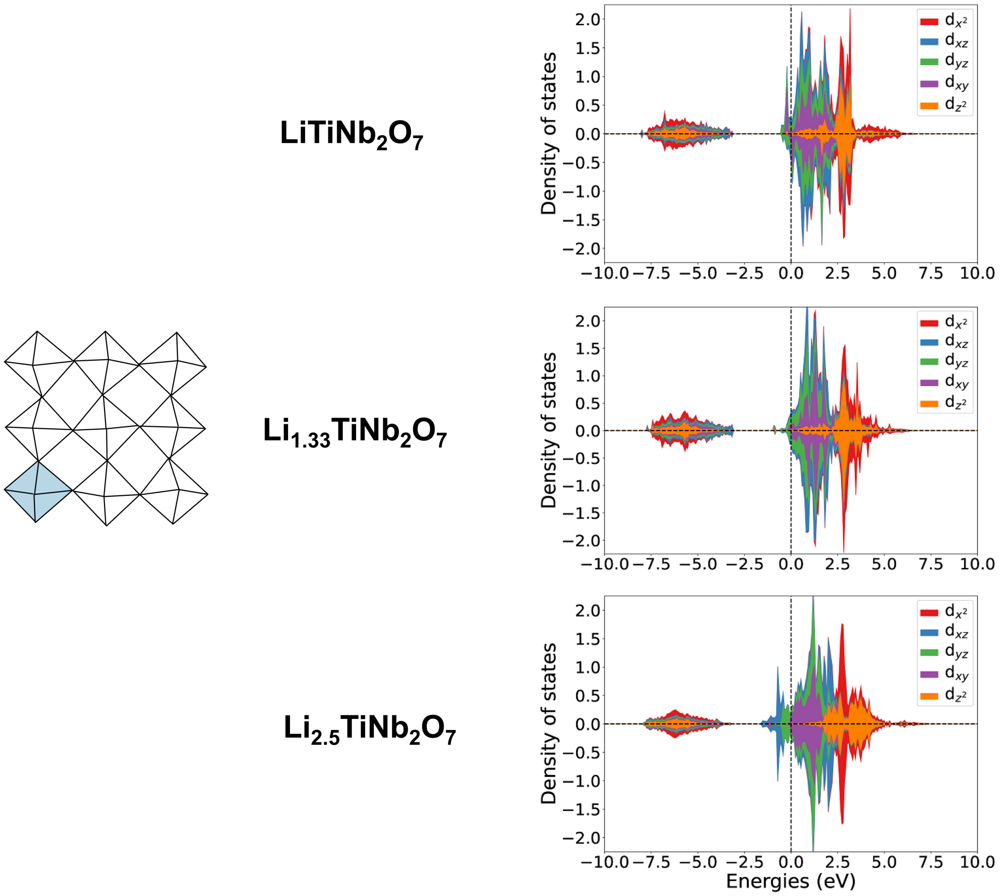

Figure S18: Projected density of states for the M2 Ti Site for $x = 1$, $x = 1.33$, and $x = 2.5$ in $Li_xTiNb_2O_7$.

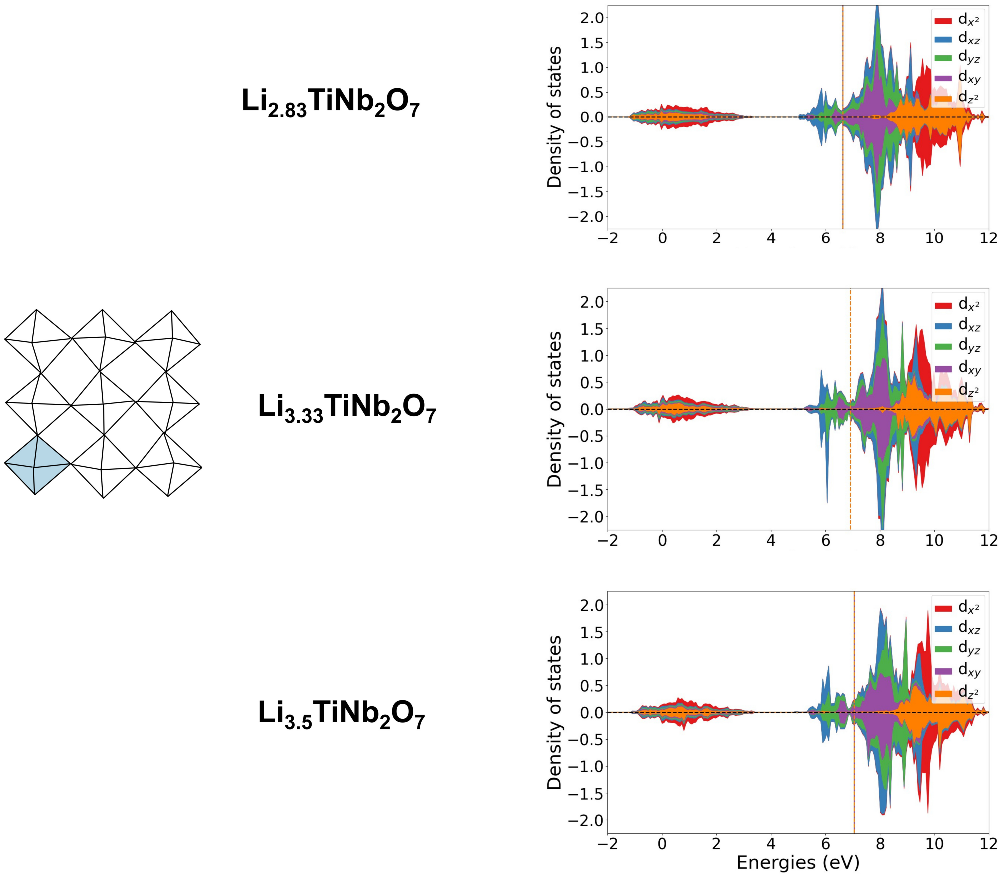

Figure S19: Projected density of states for the M2 Ti Site for $x = 2.83$, $x = 3.33$, and $x = 3.5$ in $Li_xTiNb_2O_7$.

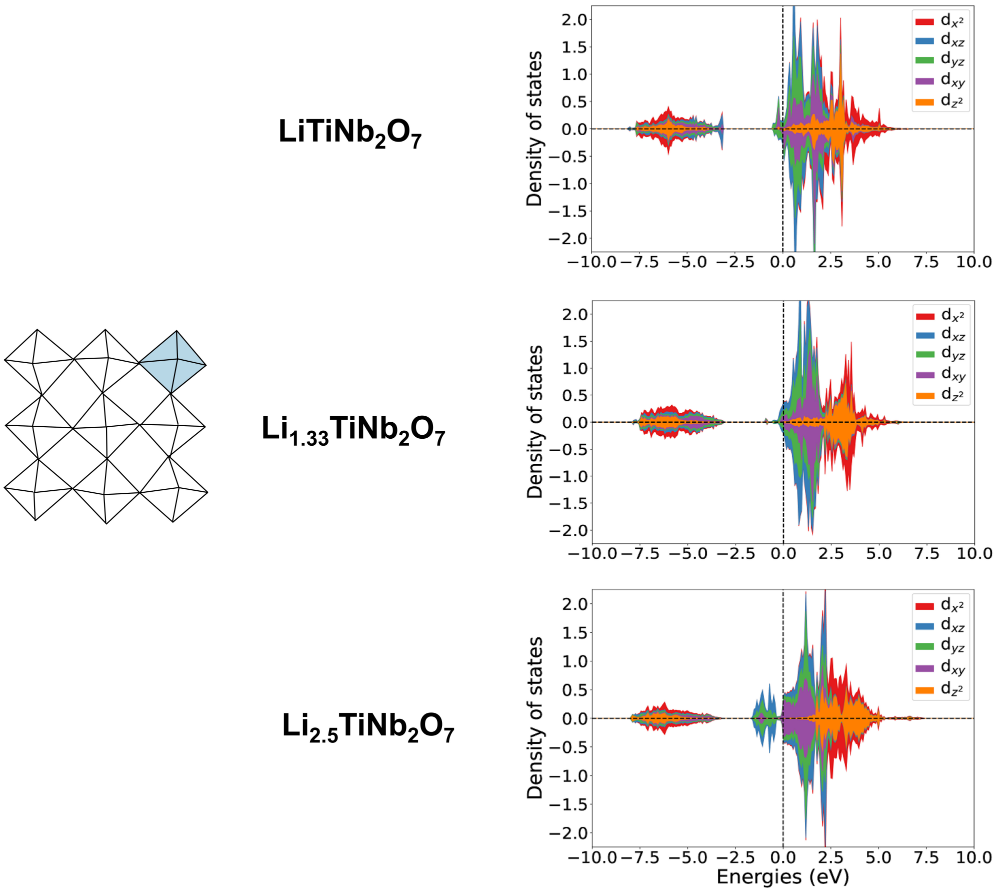

Figure S20: Projected density of states for the M2' Ti Site for $x = 1$, $x = 1.33$, and $x = 2.5$ in $Li_xTiNb_2O_7$.

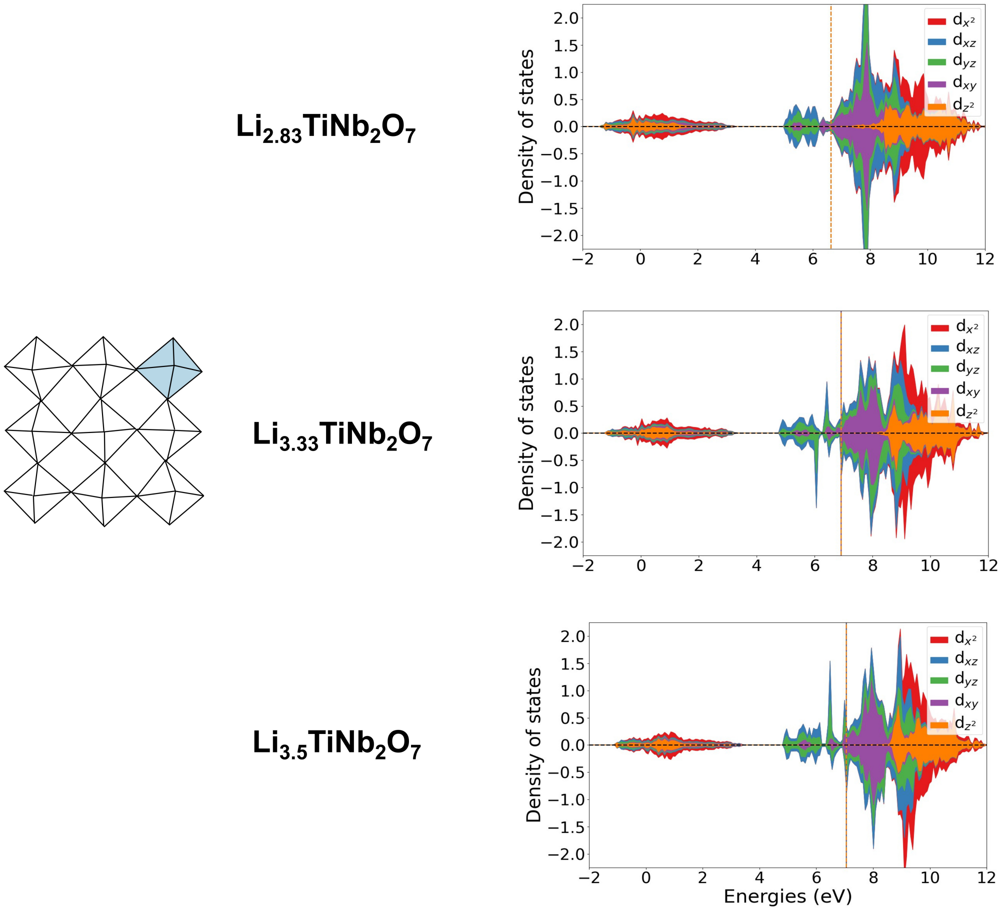

Figure S21: Projected density of states for the M2' Ti Site for $x = 2.83$, $x = 3.33$, and $x = 3.5$ in $Li_xTiNb_2O_7$.

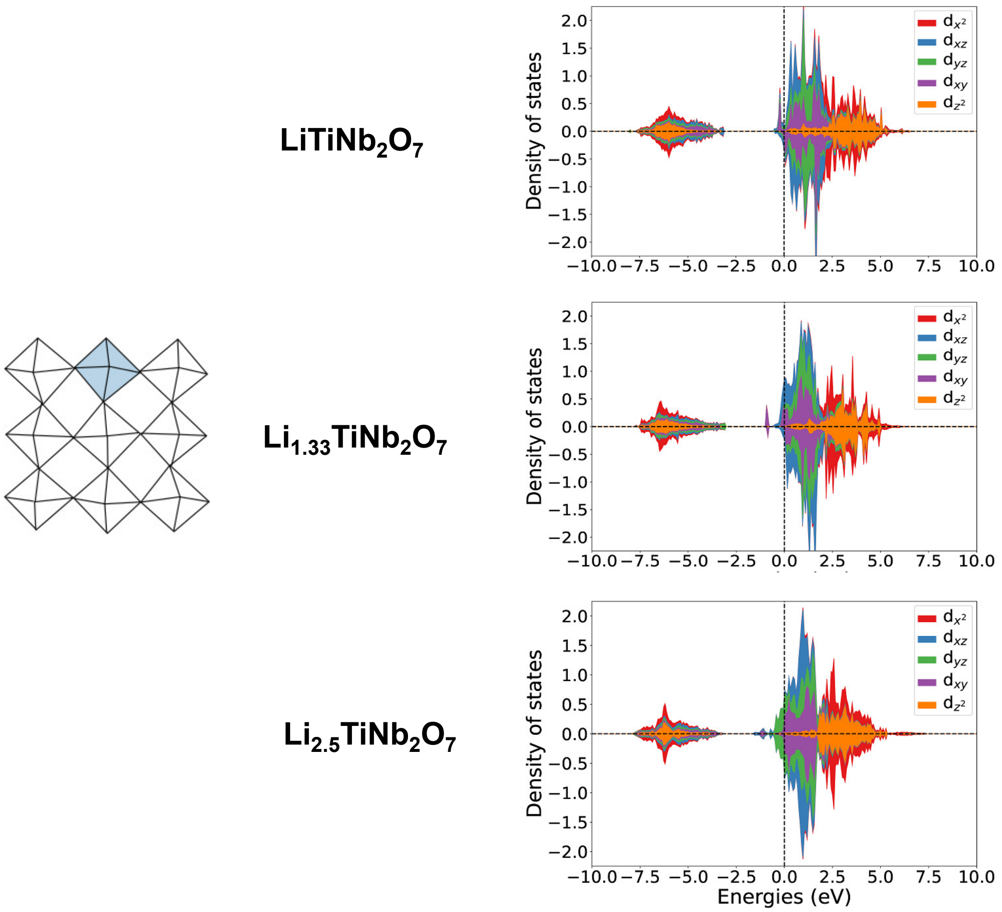

Figure S22: Projected density of states for the M3 Ti Site for $x = 1$, $x = 1.33$, and $x = 2.5$ in $Li_xTiNb_2O_7$.

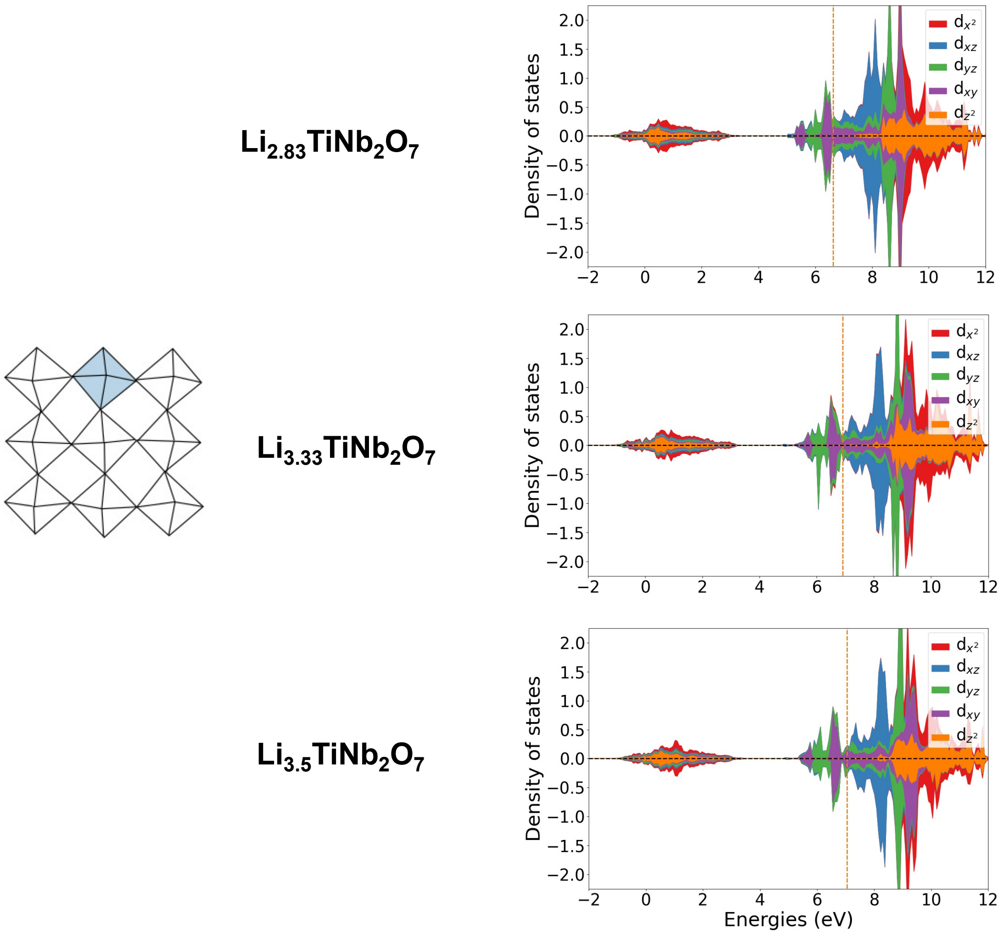

Figure S23: Projected density of states for the M3 Ti Site for $x = 2.83$, $x = 3.33$, and $x = 3.5$ in $Li_xTiNb_2O_7$.

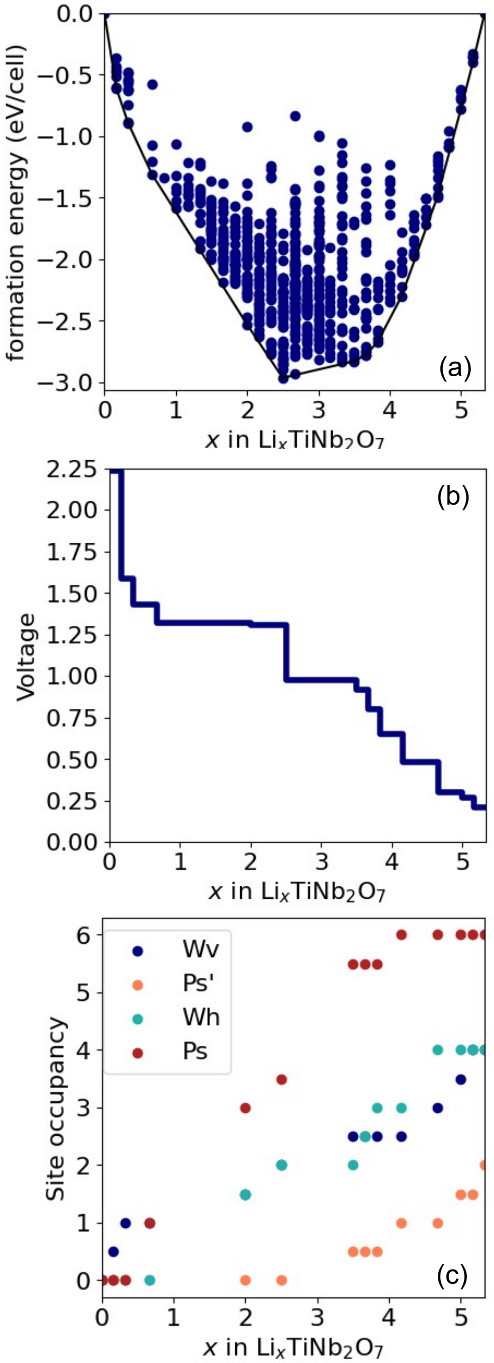

Figure S24: For the central Ti ordering of $TiNb_2O_7$ as shown in Figure S4 (b), (a) the predicted convex hull, (b) the predicted zero-temperature voltage curve upon lithiation, (c) the predicted lithium site occupancy for ground state lithium-vacancy orderings.

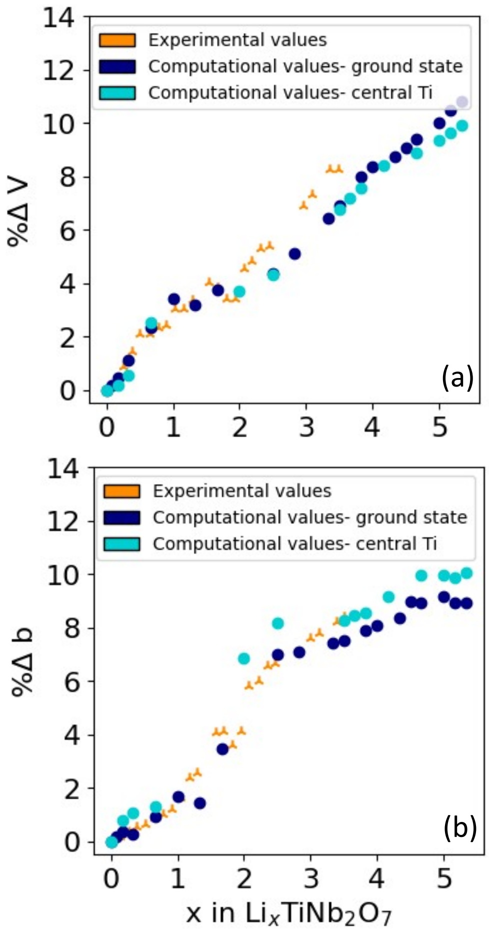

Figure S25: Percent change in volume (a) and percent change in the b lattice parameter (b) upon intercalation as predicted for the ground state ordering of $TiNb_2O_7$ (dark blue circles), the central Ti ordering of $TiNb_2O_7$ (light blue circles), and as determined from experimental $TiNb_2O_7$ samples as determined by Guo et al. [18] (orange triangles).

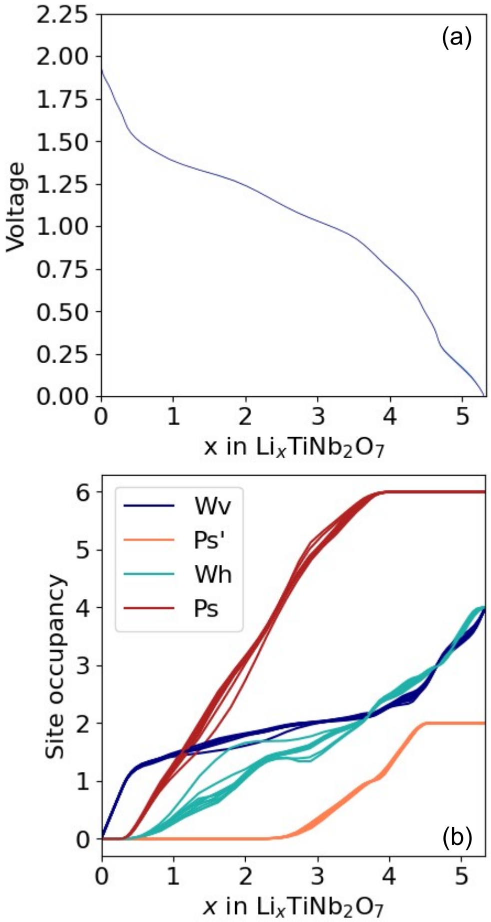

Figure S26: For the central Ti ordering of $TiNb_2O_7$ as shown in Figure S4 (b), (a) the predicted 300K voltage curve including both the mean predicted voltage and the spread as determined from ten grand canonical Monte Carlo calculations and (b) the predicted lithium site occupancy as a function of concentration over ten grand canonical Monte Carlo calculations.

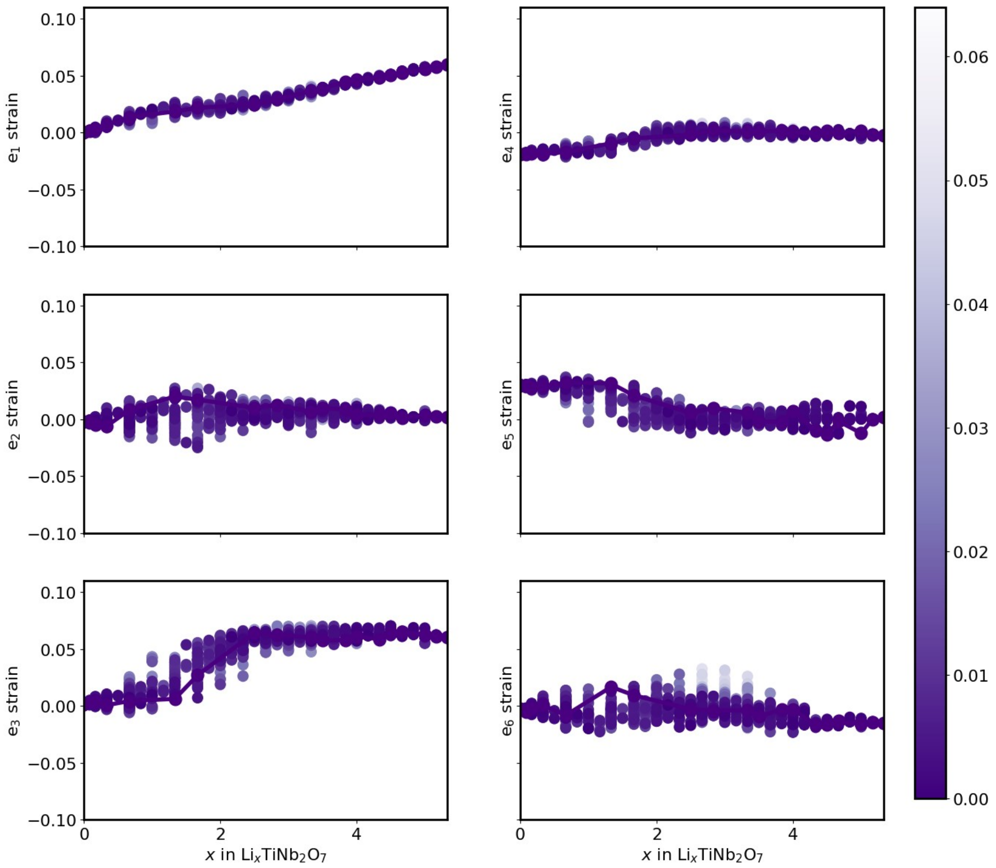

Figure S27: e_1-e_6 strain upon lithiation in $Li_xTiNb_2O_7$ for the ground state $TiNb_2O_7$ ordering.

References

1. Halasyamani, P. S. Asymmetric cation coordination in oxide materials: Influence of lone-pair cations on the intra-octahedral distortion in d^0 transition metals. *Chemistry of materials* **16,** 3586–3592 (2004).

2. Saber, M., Reynolds, C., Li, J., Pollock, T. M. & Van der Ven, A. Chemical and structural factors affecting the stability of Wadsley–Roth block phases. *Inorganic Chemistry, in press* (2023).

3. Thomas, J. C. & Van der Ven, A. Finite-temperature properties of strongly anharmonic and mechanically unstable crystal phases from first principles. *Physical Review B* **88,** 214111 (2013).

4. Thomas, J. C. & Van der Ven, A. The exploration of nonlinear elasticity and its efficient parameterization for crystalline materials. *Journal of the Mechanics and Physics of Solids* **107,** 76–95 (2017).

5. Thomas, J. C., Bechtel, J. S., Natarajan, A. R. & Van der Ven, A. Machine learning the density functional theory potential energy surface for the inorganic halide perovskite $CsPbBr_3$. *Physical Review B* **100,** 134101 (2019).

6. Bechtel, J. S., Thomas, J. C. & Van der Ven, A. Finite-temperature simulation of anharmonicity and octahedral tilting transitions in halide perovskites. *Physical Review Materials* **3,** 113605 (2019).

7. Thomas, J. C., Natarajan, A. R. & Van der Ven, A. Comparing crystal structures with symmetry and geometry. *npj Computational Materials* **7,** 164 (2021).

8. Kuhn, H. W. The Hungarian method for the assignment problem. *Naval research logistics quarterly* **2,** 83–97 (1955).

9. Kavraki, L. E. *Geometric methods in structural computational biology* (Rice University, 2009).

10. Kabsch, W. A solution for the best rotation to relate two sets of vectors. *Acta Crystallographica Section A: Crystal Physics, Diffraction, Theoretical and General Crystallography* **32,** 922–923 (1976).

11. Kabsch, W. A discussion of the solution for the best rotation to relate two sets of vectors. *Acta Crystallographica Section A: Crystal Physics, Diffraction, Theoretical and General Crystallography* **34,** 827–828 (1978).

12. Dresselhaus, M. S., Dresselhaus, G. & Jorio, A. *Group theory: Application to the physics of condensed matter* 147 ().

13. Khomskii, D. I. & Streltsov, S. V. Orbital effects in solids: basics, recent progress, and opportunities. *Chemical Reviews* **121,** 2992–3030 (2020).

14. Radin, M. D. & Van der Ven, A. Simulating charge, spin, and orbital ordering: application to Jahn–Teller distortions in layered transition-metal oxides. *Chemistry of Materials* **30,** 607–618 (2018).

15. Radin, M. D., Vinckeviciute, J., Seshadri, R. & Van der Ven, A. Manganese oxidation as the origin of the anomalous capacity of Mn-containing Li-excess cathode materials. *Nature Energy* **4,** 639–646 (2019).

16. Morita, K., Davies, D. W., Butler, K. T. & Walsh, A. Breaking the aristotype: featurization of polyhedral distortions in perovskite crystals. *Chemistry of Materials* **34,** 562–573 (2022).

17. Dreele, R. V. & Cheetham, A. K. The structures of some titanium-niobium oxides by powder neutron diffraction. *Proceedings of the Royal Society of London. A. Mathematical and Physical Sciences* **338,** 311–326 (1974).

18. Guo, B. *et al.* A long-life lithium-ion battery with a highly porous $TiNb_2O_7$ anode for large-scale electrical energy storage. *Energy & Environmental Science* **7,** 2220–2226 (2014).
